# The Immunoregulatory and Regenerative Potential of Activated Human Stem Cell Secretome Mitigates Acute-on-Chronic Liver Failure in a Rat Model

**DOI:** 10.3390/ijms25042073

**Published:** 2024-02-08

**Authors:** Barbara Cuadra, Veronica Silva, Ya-Lin Huang, Yael Diaz, Claudio Rivas, Cristobal Molina, Valeska Simon, Maria Rosa Bono, Bernardo Morales, Mario Rosemblatt, Sebastian Silva, Rodrigo Acuña, Fernando Ezquer, Marcelo Ezquer

**Affiliations:** 1 Centro de Medicina Regenerativa, Facultad de Medicina, Clínica Alemana-Universidad del Desarrollo, Av. La Plaza 680, Las Condes, Santiago 7610658, Chile; barbara.cuadra@gmail.com (B.C.); msilva01@udd.cl (V.S.); yalin1013@gmail.com (Y.-L.H.); sebastiansilva@udd.cl (S.S.); rodrigoacuna@udd.cl (R.A.); eezquer@udd.cl (F.E.); 2 Departamento de Biotecnología, Facultad de Ciencias Naturales, Matemáticas y del Medio Ambiente, Universidad Tecnológica Metropolitana, Las Palmeras 3360, Ñuñoa, Santiago 7800003, Chile; ydiaz@utem.cl (Y.D.); crivas@utem.cl (C.R.); cristobal.molinar@utem.cl (C.M.); 3 Departamento de Biología, Facultad de Ciencias, Universidad del Chile, Las Encinas 3370, Ñuñoa, Santiago 7800020, Chile; valeskasimon@yahoo.es (V.S.); mrbono@uchile.cl (M.R.B.); 4 Facultad de Ciencias de la Salud, Universidad del Alba, Atrys Chile, Guardia Vieja 339, Providencia, Santiago 7510249, Chile; labdrmorales@gmail.com; 5 Centro de Ciencia & Vida, Av. Del Valle Norte 725, Huechuraba, Santiago 8580702, Chile; mrosemblatt@cienciavida.cl

**Keywords:** mesenchymal stem cells, in vitro preconditioning, acute-on-chronic liver failure, secretome, multiorgan failure

## Abstract

Acute-on-chronic liver failure (ACLF) is a syndrome marked by sudden liver function decline and multiorgan failure, predominantly acute kidney injury (AKY), in patients with chronic liver disease. Unregulated inflammation is a hallmark of ACLF; however, the key drivers of ACLF are not fully understood. This study explores the therapeutic properties of human mesenchymal stem cell (MSC) secretome, particularly focusing on its enhanced anti-inflammatory and pro-regenerative properties after the in vitro preconditioning of the cells. We evaluated the efficacy of the systemic administration of MSC secretome in preventing liver failure and AKI in a rat ACLF model where chronic liver disease was induced using by the administration of porcine serum, followed by D-galN/LPS administration to induce acute failure. After ACLF induction, animals were treated with saline (ACLF group) or MSC-derived secretome (ACLF-secretome group). The study revealed that MSC-secretome administration strongly reduced liver histological damage in the ACLF group, which was correlated with higher hepatocyte proliferation, increased hepatic and systemic anti-inflammatory molecule levels, and reduced neutrophil and macrophage infiltration. Additionally, renal examination revealed that MSC-secretome treatment mitigated tubular injuries, reduced apoptosis, and downregulated injury markers. These improvements were linked to increased survival rates in the ACLF-secretome group, endorsing MSC secretomes as a promising therapy for multiorgan failure in ACLF.

## 1. Introduction

Acute-on-chronic liver failure (ACLF) is a complex clinical syndrome, characterized by acute hepatic failure in patients with pre-existing chronic liver disease [1,2]. This syndrome exhibits high mortality rates, which can reach 50–80% within 28 days of the onset of the disease [3]. The most common etiologies for pre-existing chronic conditions include cirrhosis due to alcoholism and hepatitis B virus (HBV) infection. In comparison, bacterial infections, detected in two-thirds of the patients, are the most frequent inductors of acute failure and significantly contribute to the poor outcome of ACLF patients [4,5]. Interestingly, mortality in ACLF appears to be independent of the presence or type of precipitating event [2,6].

The precise mechanisms initiating ACLF remain unclear, but unregulated inflammation is widely accepted as a key contributing factor [7,8]. It has been reported that the combination of chronic hepatic injury, with both innate and adaptive local inflammatory responses, and enhanced oxidative stress, perpetuates a vicious cycle, escalating local hepatic inflammation into a systemic inflammatory response [9]. Once ACLF is established, the severity of the inflammatory process is believed to be crucial in determining the short-term prognosis and the syndrome’s progression [6,10].

Reports in ACLF patients also suggest that hepatocyte proliferation, which is the primary regenerative response in mild-to-severe hepatic injury, is markedly impaired in end-stage ACLF [11]. Consequently, patients who survive the initial phase of ACLF often present an extended period of convalescence and recovery, marked by sequelae associated with persistent multiorgan failure, leading to a generally poor prognosis [12,13]. Additionally, the systemic spread of inflammation to other organs disrupts cellular function and can lead to necrosis and apoptosis. Thus, multiorgan compromise is a major contributor to the increased mortality associated with ACLF. In this sense, the kidneys are particularly susceptible to the systemic changes seen in ACLF [14]. Therefore, the regulation of the immune response and the promotion of liver cell regeneration are crucial goals in the development of new therapeutic strategies for this disease.

Nowadays, treatment for ACLF is predominantly supportive, with no specific therapies available. Liver transplantation remains the only proven beneficial treatment, but its accessibility is limited by the rapid progression of the disease and the scarcity of donors [10,15]. Despite the absence of a universal consensus regarding the pathophysiological definition of ACLF, all multicenter studies agree that it is a devastating syndrome, with exceptionally high mortality, presenting one of the most significant challenges in hepatology [1]. Hence, there is an urgent need to explore new treatment options to improve the quality of life and survival rates of ACLF patients.

Mesenchymal stem cells, also known as multipotent mesenchymal stromal cells (MSCs), are a population of self-renewable and undifferentiated cells present in multiple mesenchymal tissues, including bone marrow, umbilical cord, dental pulp, and adipose tissues. Recognized for their extensive anti-inflammatory, antioxidant, and pro-regenerative properties, MSCs are becoming a promising therapeutic option for complex diseases such as ACLF. These effects are mediated through both the paracrine secretion of trophic factors and direct cell-to-cell contact. In fact, MSCs are increasingly considered the body’s “drug store” due to their ability to create a pro-regenerative microenvironment, which is considered to be their primary therapeutic mechanism [16,17].

It is widely recognized that, in contrast to traditional pharmacological treatments, MSCs operate through multiple pathways. They release a wide spectrum of growth factors, such as BMP4, bFGF, EGF, and HGF, which support progenitor cell survival, enhance hepatocyte proliferation, stimulate revascularization, and inhibit the proliferation of hepatic stellate cells (HSCs), which are responsible for hepatic fibrosis [18,19]. However, MSCs´ most distinctive feature is their modulation of both the innate and adaptive immune systems, amplifying anti-inflammatory pathways within the damaged hepatic milieu. This response is initiated through the secretion of soluble factors, including PGE2, IL-10, IDO, TSG-6, and TGFb [20].

Studies in animal models have shown that MSC treatment is effective in enhancing liver function [21,22], ameliorating liver fibrosis [23], and reversing fulminant hepatic failure [24], and the safety profile of MSC-based therapies is well established, with hundreds of clinical trials reporting no serious adverse events. However, the remarkable success observed in preclinical studies has yet to be fully replicated in clinical settings [25,26]. In this sense, different reports suggest that MSC treatment improves liver function in patients with chronic liver diseases of various etiologies [27,28], although some studies have not observed significant benefits [29].

Recently, diverse clinical trials have been conducted to assess the impact of MSCs on ACLF, but inconsistencies in research design and evaluation criteria make comparisons challenging. Liu et al. performed a meta-analysis of randomized clinical trials using MSCs for ACLF and revealed that MSC administration moderately enhances hepatic recovery without impacting 48-week patient survival rates [30]. In contrast, Shi et al. found no significant differences in 13-month survival rates for ACLF patients treated with MSCs but noted improved survival rates 13–75 months after treatment [31].

Diverse factors could affect the in vivo effectiveness of MSCs. Post-administration, MSCs migrate to injured tissues, where the presence of a harsh environment and inadequate cell–matrix tensegrity can impede their efficacy, thus affecting their survival [32,33,34,35]. Moreover, the immunosuppressive capacity of MSCs is not inherent but requires activation by the local microenvironment [36]. This evidence highlights the necessity for optimizing cell-based therapies, potentially by modifying cell manufacturing processes or incorporating specific cell preconditioning strategies before transplantation [37,38,39].

Recent findings reveal that the in vitro preconditioning of MSCs with pro-inflammatory cytokines strongly increases their anti-inflammatory, pro-regenerative, and antioxidative properties. This enhancement is achieved through the increased secretion of anti-inflammatory and immunomodulatory factors [37,40,41,42], and thus they emerge as a potent therapeutic tool against inflammatory diseases. In this way, it has been shown that MSCs’ therapeutic effects are largely due to a transient paracrine impact, involving the release of soluble factors and extracellular vesicles constituting the secretome [21,43,44,45]. Consequently, there is growing interest in using MSC-derived secretomes rather than living cells. The MSC secretome, which can be lyophilized for easier storage and use, is subject to safety, dosage, and potency assessments akin to conventional pharmaceuticals, offering a viable option for diseases like ACLF with restricted therapeutic windows [46,47,48].

Considering that the secretome is expected to replicate the multifaceted effects of MSC, this study focuses on evaluating the therapeutic effects of the systemic administration of the secretome derived from MSCs in an animal model of severe ACLF [49,50]. This secretome is obtained from human MSCs that have been preconditioned in vitro with TNF-α and INFγ. The objective of this preconditioning process is to enhance the therapeutic potential of the secretome for reducing hepatic damage, which is associated with both hepatic and systemic inflammation, as well as oxidative stress. A complimentary goal of this study is to evaluate the potential of the secretome in preventing damage to extra-hepatic organs, such as the kidneys, which is a common complication in ACLF patients, finally improving the survival rate in the evaluated experimental model.

## 2. Results

### 2.1. MSC-Secretome Administration Increases Survival Rate and Attenuates ACLF Hepatic Injury

To generate the ACLF animal model, PS was administered biweekly over 11 weeks, resulting in immune-mediated liver cirrhosis, representing the chronic component of the disease [49,50]. A subset of animals was euthanized after chronic injury induction (cirrhosis group) to evaluate the histopathological alterations in the liver following PS administration (Figure S1). Livers from control animals exhibited clear structure, uniform hepatocyte sizes, and the absence of microarchitectural distortions or necrosis. In contrast, livers from SP-treated animals demonstrated bile duct proliferation, mild inflammatory cell infiltration, pseudolobule formation, and fibrotic septum development, confirming the onset of fibrosis/cirrhosis. None of these animals (cirrhosis group) died during the experimental period.

**Figure 1:**
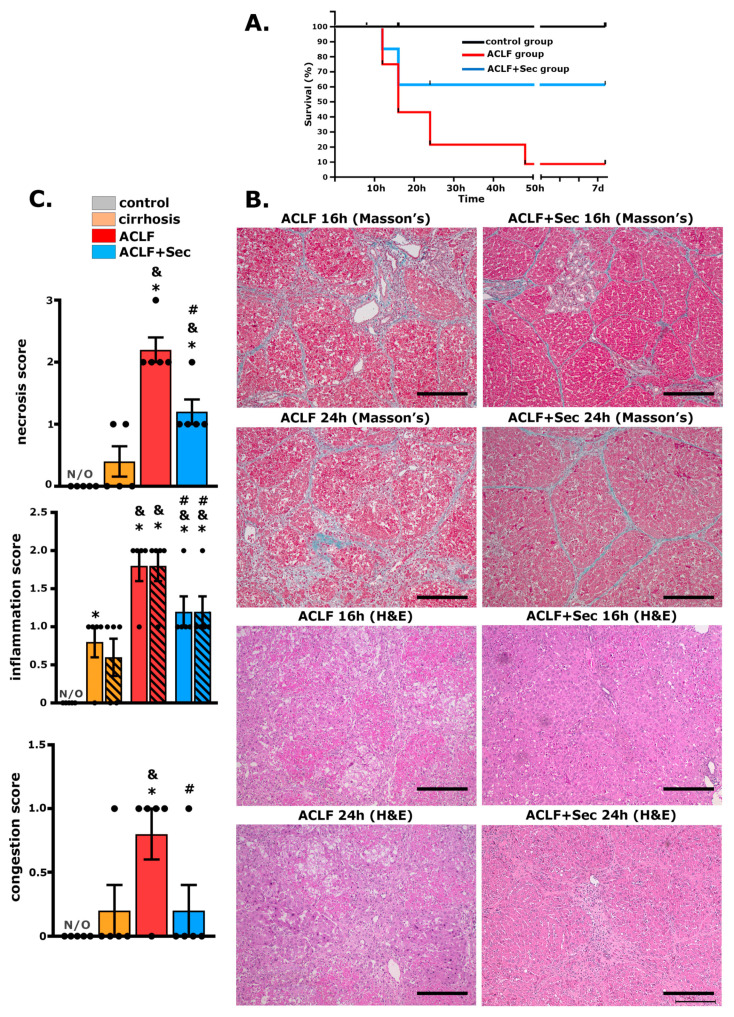
*MSC-Secretome administration enhances survival rate and attenuates ACLF hepatic injury.* To establish the ACLF model, rats were exposed to porcine serum administration for 11 weeks (cirrhosis development), followed by the administration of LPS/DGalN as an acute challenge to induce ACLF. Survival rates and hepatic damage were assessed up to 7 days post-acute challenge: (**A**) Kaplan–Meier survival analysis was carried out for control, ACLF, and ACLF + Sec groups; *n* = 10 in the control group, *n* = 35 in ACLF and ACLF + Sec groups. (**B**) Masson’s trichrome and H&E staining were performed to evaluate MSC-secretome effects in the ACLF model. Representative micrographs show liver histology of ACLF rats treated with the vehicle (ACLF group) and ACLF rats treated with the MSC secretome derived from 1 × 10^6^ in vitro preconditioned MSCs (ACLF + Sec), 16 and 24 h after ACLF induction (scale bars represent 250 µm). (**C**) Scoring of necrosis, periportal inflammation (solid bar), pericentral inflammation (lined bar), and congestion 24 h post-acute injury. N/O = not observed. Data are presented as mean ± SEM, whereas dots represet individual values, *n* = 5; * *p* < 0.05 vs. control group; & *p* < 0.05 vs. cirrhosis group; # *p* < 0.05 vs. ACLF group.

After 11 weeks of PS exposure, rats with liver fibrosis were treated with LPS/D-GalN to induce acute injury and generate the ACLF model [49,51,52,53,54]. Follow-up was conducted seven days post-ACLF induction. The locomotor activity level in these rats significantly declined, indicating severe health deterioration. Consistent with previous studies, 80% of the animals in the ACLF group died within the first 16–24 h [49,50]. This high rate of short-term mortality aligns with the known clinical and pathological features of ACLF. At the 7-day post-LPS/DGalN administration, the survival rate for this experimental group was reduced to only 10% (Figure 1A).

In an initial experimental evaluation, a subset of the ACLF animals was treated with the secretome derived from 1 × 10^6^ non-preconditioned MSCs, administered 60 min following the LPS/D-GalN challenge. This intervention did not significantly improve survival rates, reaching 30% at the end of the 7-day follow-up period (Figure S2).

It is well known that the in vitro preconditioning of MSCs can markedly enhance their anti-inflammatory, antioxidative, and pro-regenerative properties [37]. Therefore, another group of ACLF animals was treated with the same secretome dose but derived from MSCs that were preconditioned with TNF-α and INFγ in vitro. In this case, most deaths still occurred within 16–24 h post-LPS/DGalN challenge; however, the 7-day survival rate in the ACLF + Sec group increased significantly, reaching 60% (Figure 1A).

Considering these outcomes, subsequent studies exclusively employed the secretome derived from preconditioned MSCs.

After the animals were euthanized, the hepatic, renal, and spleen indices (organ weight/body weight) were determined. Data indicate that the liver index in the ACLF group was significantly increased at 16 h after the challenge compared to the control and cirrhosis groups (Table 1). Similarly, the cirrhosis group exhibited an increase in the spleen index compared to the control group, reflecting immune-mediated liver fibrosis. This index increased dramatically in the ACLF group from 8 to 24 h, whereas the ACLF + Sec group showed a significant decrease in both liver and spleen indices (Table 1).

At 8 h post-ACLF induction (LPS/DGalN administration), ballooning and the degeneration of hepatocytes were observed, along with areas of necrosis. However, from 16 to 24 h after the challenge, in line with increased animal mortality, livers in the ACLF group exhibited heightened hepatocellular necrosis, pronounced septal fibrosis, hepatic congestion, cytoplasmic vacuolization, and severe distortion of tissue architecture (Figure 1B). In contrast, livers from the ACLF + Sec group demonstrated reduced hepatocyte death, less edema, and thinner septal fibrosis.

Additionally, a significant influx of inflammatory cells was observed in the liver of ACLF rats. In contrast, the rats treated with the secretome displayed fewer liver infiltrating inflammatory cells (Figure 1B). For semiquantitative histological examination, a blinded specialist pathologist determined necrosis, congestion, and inflammatory scores, using previously validated scoring systems [55,56]. The ACLF + Sec group exhibited lower individual scores than the vehicle-treated ACLF group (Figure 1C).

Seven days after the induction of acute failure, both the ACLF and ACLF + Sec groups showed recovery in hepatic lobular microarchitecture alterations, a significant reduction in inflammatory activity, and the disappearance of apoptosis. However, some regenerative nodules persisted in the secretome-treated group (Figure S3).

Gluconeogenesis in hepatocytes (observed via PAS staining) is a reliable marker of functional cell state. This marker significantly decreased during the acute phase of the challenge (24 h) (95% ± 1.9 in the control group; 60% ± 5.8 in the cirrhosis group; 3.9% ± 0.6 in the ACLF group, and 8 ± 0.9 in the ACLF + Sec group). However, a recovery rate close to normal values was observed at 7 days, coinciding with hepatic regeneration in the surviving animals.

Liver function in each experimental group was assessed through serological tests. As indicated in Table 2, both transaminases, AST and ALT, and alkaline phosphatase significantly increased in the ACLF and ACLF + Sec groups between 8 and 24 h post-ACLF induction. However, secretome administration resulted in a partial decrease in AST levels at 16 h. Similarly, direct bilirubin levels, associated with biliary secretion disorders and changes in biliary duct permeability, showed a significant increase between 8 and 24 h post-ACLF induction in the ACLF group and a significant reduction in the secretome-treated animals. The plasma levels of all evaluated markers returned to near-normal values 7 days after the induction of ACLF.

### 2.2. MSC-Secretome Administration Reduces the Apoptotic Rate and Induces Hepatocyte Proliferation after ACLF Induction

**Figure 2:**
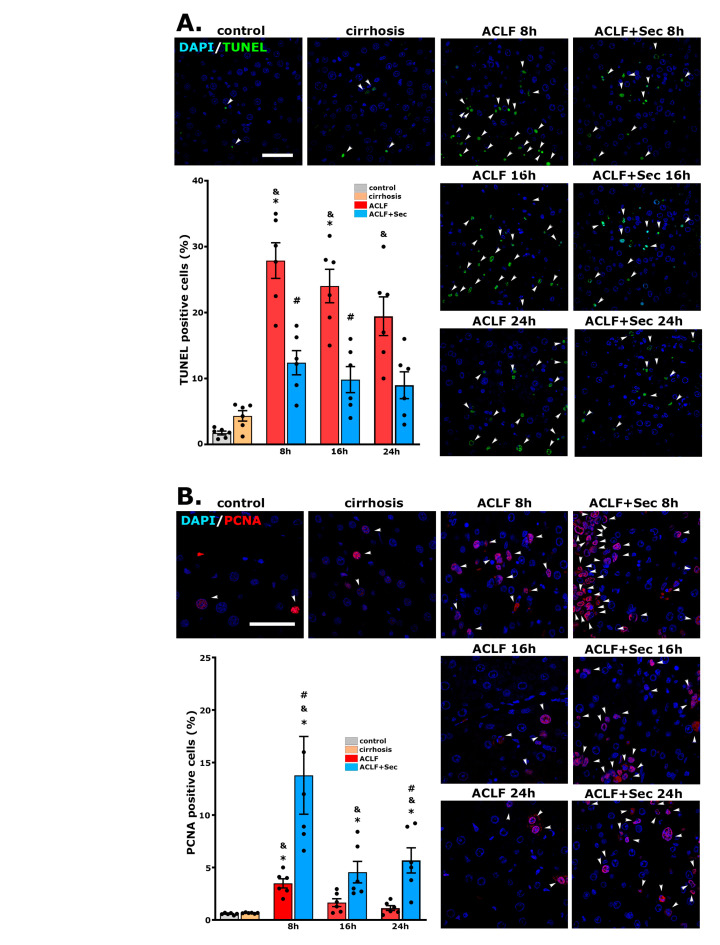

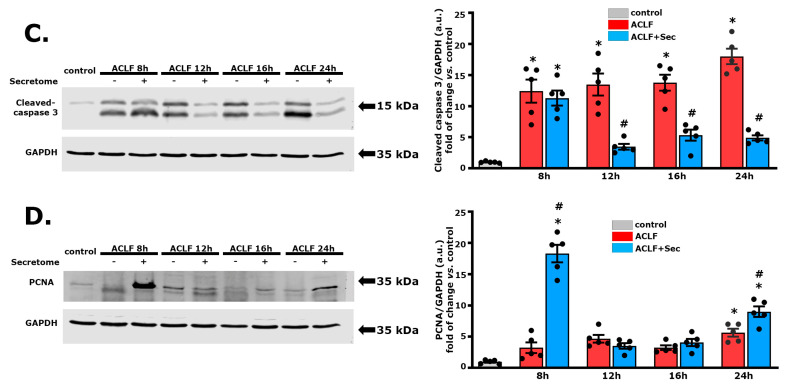
*MSC-secretome administration reduces apoptotic rate and promotes hepatocyte proliferation after ACLF induction.* Apoptosis and cellular proliferation were analyzed at 8, 16, and 24 h post-ACLF induction in all experimental groups. MSC secretome’s effect on apoptosis was assessed via TUNEL staining (FITC—green), and proliferation was assessed via PCNA immunoreactivity (Alexa Fluor 555—red); in both cases, the nuclei were counterstained with DAPI (blue): (**A**) Representative micrographs of cell apoptosis determined by TUNEL (white arrowheads). (**B**) Representative micrographs of hepatocyte proliferation determined by PCNA labeling (white arrowheads). Quantification of positive nuclei per 100 hepatic cells was carried out using digital imaging. Data represent mean ± SEM for 30 fields/animal, six animals/group. Scale bars represent 50 µm. Complementary semiquantitative analysis of apoptosis and proliferation was performed via Western blotting for (**C**) cleaved caspase 3 and (**D**) PCNA in liver tissue 8, 12, 16, and 24 h post-ACLF induction. Protein levels were normalized against GAPDH. Data are presented as mean ± SEM, whereas dots represet individual values, *n* = 5; * *p* < 0.05 vs. control group; & *p* < 0.05 vs. cirrhosis group; # *p* < 0.05 vs. ACLF group.

As previously indicated, the chronic hepatic condition (cirrhosis) renders hepatocytes particularly vulnerable to injuries, which is associated with the inhibition of the endogenous regenerative capacity [11,12]. Since hepatocyte death occurs if cells cannot complete the mitotic cycle, the TUNEL assay was employed to investigate whether apoptosis contributed to the failure in liver regeneration in ACLF animals. As illustrated in Figure 2A, the TUNEL assay revealed scarce apoptotic cells in the control group, while animals in the cirrhosis group did not exhibit a significant increase. However, the rats in the ACLF groups displayed an elevated apoptotic rate between 8 and 24 h after acute failure initiation. Conversely, a significant reduction in TUNEL (+) nuclei was observed in the livers of MSC-secretome-treated animals (Figure 2A).

These findings were corroborated by assessing the immunoreactivity of cleaved caspase 3 in hepatic tissue through Western blotting. In agreement with previous results, no differences were found in the apoptotic markers between the control and cirrhosis groups (Figure S4). However, the induction of acute failure led to an increase in cleaved caspase 3 immunoreactivity, while secretome administration resulted in a significant decrease that could be evidenced after 12 h of the ACLF induction (Figure 2C).

Immunofluorescence staining for the proliferation marker PCNA was developed to evaluate hepatic proliferation activity. As shown in Figure 2B, acute injury induction triggered a proliferative response, which was significantly enhanced in the ACLF group treated with secretome, at least up to 24 h after ACLF induction. Complementarily, we assessed PCNA immunoreactivity in hepatic tissue by Western blotting. No significant differences were found between the control and cirrhosis groups (Figure S4). However, secretome administration increased total PCNA immunoreactivity at 8 and 24 h post-acute challenge compared with the ACLF vehicle-treated group (Figure 2D). Previous studies have shown that once the regenerative response in the liver is induced, an early response in PCNA expression can be observed, followed by a second stimulus of proliferation 24–48 h later [57]. The lack of a correlation between PCNA values determined by immunofluorescence and Western blotting could be related to the fact that, in immunofluorescence evaluation, there is no threshold for immunoreactivity, and nuclei with high or low PCNA expression are considered positives [58]. On the other hand, Western blotting is a semiquantitative technique representing the total amount of PCNA in the tissue. For this reason, in the current study, both methodologies were used in a complementary way. It is important to highlight that, with both methods, it was observed that the administration of secretome had an inducing effect on hepatic regeneration.

Collectively, these results demonstrate that the systemic administration of secretome in this ACLF model reduces hepatocellular death and induces hepatocyte proliferation.

### 2.3. MSC-Secretome Administration Increases Anti-Inflammatory Cytokine Expression

ACLF is characterized by a complex disbalance in the hepatic and systemic immune responses [8]. We evaluated the expression of key cytokines in the liver to characterize the ACLF animal model and the effects of MSC-secretome administration.

**Figure 3:**
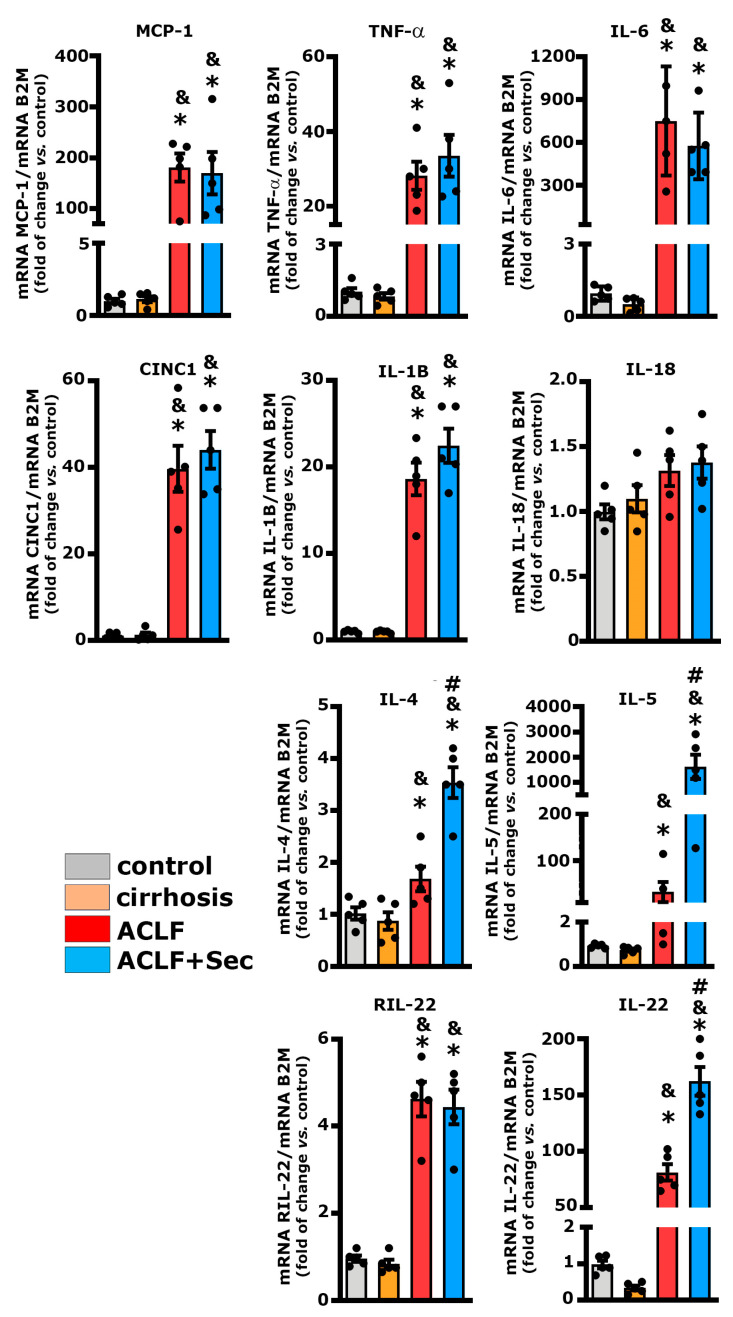
MSC-secretome administration increases hepatic anti-inflammatory cytokine expression after ACLF induction. The hepatic mRNA levels of the cytokines MCP-1, TNF-α, IL-6, CINC1, IL-1β, IL-18, IL-4, IL-5, IL-22, and IL-22 were quantified by RT-qPCR 16 h post-ACLF induction (LPS/DGalN administration), normalized against β2 microglobulin (B2M) and expressed as fold change vs. control group. Data are presented as mean ± SEM, whereas dots represet individual values (*n* = 5) (* *p* < 0.05 vs. control group; & *p* < 0.05 vs. cirrhosis group; # *p* < 0.05 vs. ACLF group).

In both the ACLF and ACLF + Sec groups, there was a rapid increase (within 16 h post-acute failure induction) in the hepatic expression levels of pro-inflammatory cytokines such as MCP-1, TNF-α, IL-6, CINC-1, and IL-1β (Figure 3). This response is linked to the severity of the ACLF model used, which is associated with an exacerbated inflammatory response.

Although the administration of MSC-derived secretome did not decrease the expression levels of the evaluated pro-inflammatory cytokines, a significant increase in the expression of cytokines with strong anti-inflammatory properties, such as IL-4 and IL-5, was observed (Figure 3).

It has been previously reported that IL-22 plays a crucial role in stimulating the proliferative potential of hepatocytes under pathological conditions like ACLF [59]. In this study, both the ACLF and ACLF + Sec groups showed an increase in the expression of the IL-22 receptor (RIL-22). However, only the group treated with secretome exhibited a significant increase in IL-22 expression (Figure 3).

The exacerbated immune response was confirmed by quantifying the protein levels of pro- and anti-inflammatory molecules using the Milliplex system, at 16 h, 24 h, and 7 days post-acute failure induction, both in hepatic tissue and plasma (Table 3). Consistent with the RT-qPCR findings, an increase in hepatic pro-inflammatory cytokine levels was observed in both experimental groups (ACLF and ACLF + Sec). However, the MSC-secretome-treated animals exhibited a partial decrease in hepatic TNF-α, CINC-1, and IL-6 levels compared to the ACLF vehicle-treated group. Additionally, the ACLF + Sec group showed higher levels of the anti-inflammatory molecules IL-2, IL-10, IL-13, IL-4, and IL-5 (Table 3).

In agreement with the previous findings, a significant increase in the plasma levels of pro-inflammatory cytokines was observed in both ACLF and ACLF + Sec groups at 16 and 24 h after acute liver failure. Conversely, animals treated with the secretome exhibited elevated levels of anti-inflammatory molecules such as IL-2, IL-10, IL-13, IL-4, and IL-5, primarily noted at 16 h following the induction of acute injury. Seven days after the acute challenge, most of the inflammatory markers returned to normal values, aligning with the resolution of hepatic failure observed in the surviving animals.

Consistent with observations in ACLF patients, the inflammatory response extended beyond hepatic tissue in the experimental model utilized, demonstrating a systemic general response [8,9]. The present results suggest that secretome administration induces an increase in the anti-inflammatory response and, to a lesser extent, a reduction in the pro-inflammatory response during a critical period associated with the survival of animals with ACLF (16 h after the onset of ACLF induction).

### 2.4. MSC-Secretome Administration Decreases Hepatic Neutrophil and Macrophage Infiltration

**Figure 4:**
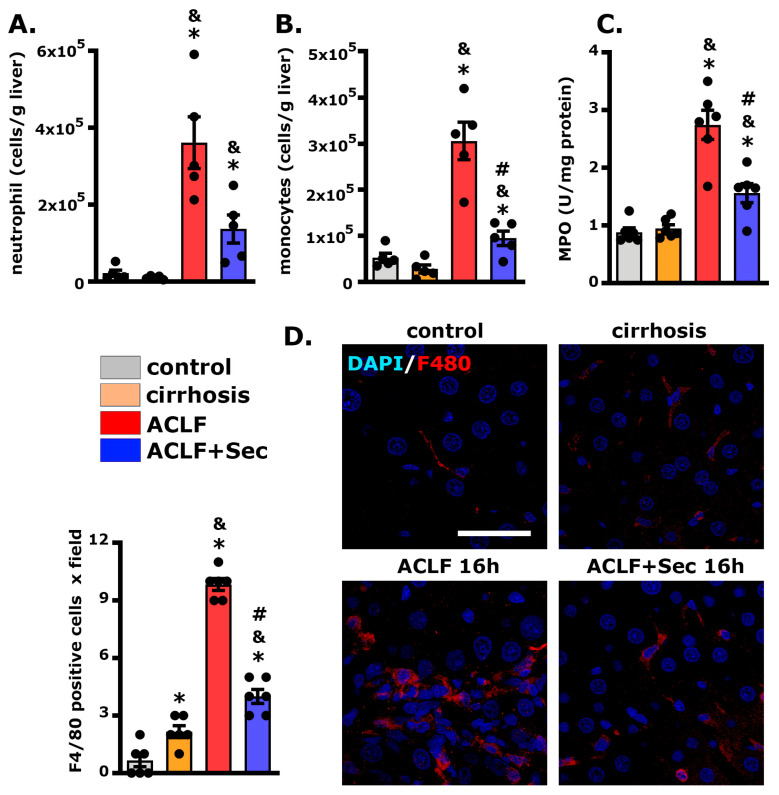
*MSC-secretome administration decreases hepatic neutrophil and macrophage infiltration after ACLF induction.* Hepatic infiltration of (**A**) neutrophils (labeled with RP1) and (**B**) monocytes (labeled with His48) were evaluated by flow cytometry 16 h post-ACLF induction, *n* = 4. (**C**) Myeloperoxidase (MPO) levels in hepatic tissues reflect neutrophil infiltration; data were normalized to protein amount and expressed as fold change vs. control group, *n* = 6. (**D**) Macrophage infiltration (F4/80 Alexa 555, red) in hepatic tissue 16 h post-ACLF induction was assessed by confocal microscopy. Nuclei were counterstained with DAPI (blue). Scale bars represent 50 µm. Quantification of positive cells was carried out by digital imaging analysis. Data are presented as mean ± SEM for 30 fields/animal, six animals/group, whereas dots represet individual values; * *p* < 0.05 vs. control group; & *p* < 0.05 vs. cirrhosis group; # *p* < 0.05 vs. ACLF group.

Neutrophil infiltration into the liver constitutes a pivotal event in ACLF, reflecting a sustained systemic inflammatory response within the hepatic milieu [60,61]. Flow cytometric analysis revealed a significant increase in hepatic neutrophil infiltration in the ACLF group 16 h post-acute injury compared with both the control and cirrhosis groups. However, MSC-secretome administration resulted in a substantial reduction in neutrophil infiltration (*p* = 0.058) (Figure 4A and Figure S5). In addition, we evaluated the hepatic infiltration of monocytes by flow cytometry and obtained similar results, highlighting the decrease in the inflammatory infiltration resulting from the MSC-secretome administration (Figure 4B and Figure S5). To complement the flow cytometric analysis, the hepatic activity of myeloperoxidase (MPO), the most abundant protein in neutrophils, was assessed at the same experimental point [62]. Consistent with the previous findings, the ACLF group exhibited an increase in MPO activity compared to the control and cirrhosis groups. In contrast, secretome administration led to a significant decrease in the activity of this enzyme (Figure 4C).

Additionally, we employed confocal microscopy to evaluate macrophage infiltration in the liver 16 h post-acute injury. As shown in Figure 4D, the number of F4/80(+) cells per field was significantly higher in the ACLF group than in the control and cirrhosis groups. Meanwhile, the administration of secretome induced a significant reduction in the number of macrophages per field.

These observations align with the previous results, suggesting that administration of MSC secretome diminishes hepatic inflammatory cell infiltration in the ACLF model.

### 2.5. MSC-Secretome Administration Increases Hepatic Nrf2 and Heme Oxygenase Levels While Decreasing DNA Oxidative Damage

Nuclear factor erythroid 2 p45-related factor 2 (Nrf2) is associated with cytoprotective gene expression such as heme oxygenase 1 (HO-1) [63]. Both factors are linked to a reduction in oxidative stress and the stimulation of hepatocyte proliferation in response to injury [64].

**Figure 5:**
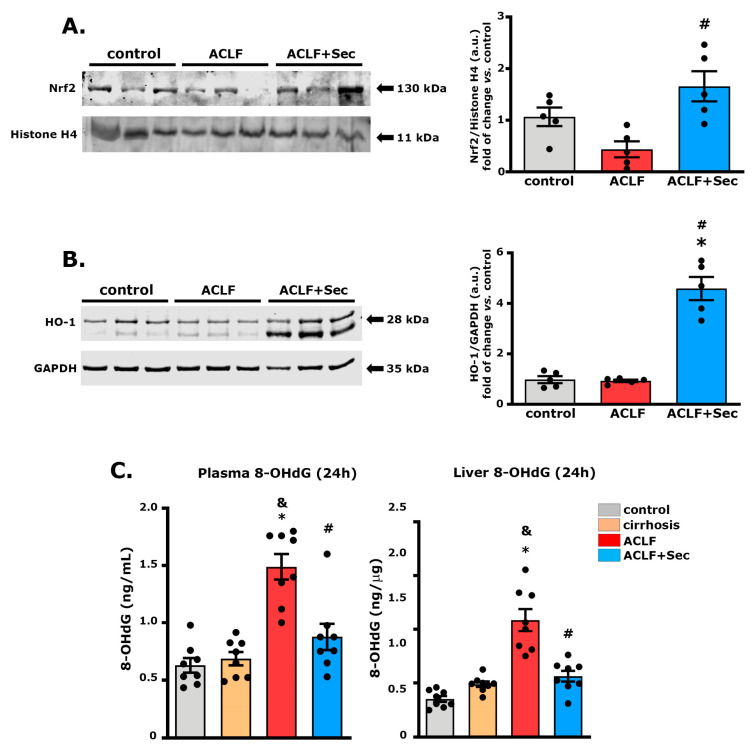
*MSC-secretome administration increases hepatic Nrf2 and HO-1 levels, decreasing oxidative damage after ACLF induction:* (**A**) Nrf2 levels were quantified in the nuclear fraction of hepatic tissue 16 h post-ACLF induction. Values were normalized against histone H4. (**B**) Heme oxygenase 1 (HO-1) levels were determined in total liver tissue fraction at the same time point, and data were normalized against GAPDH. Results for both markers are presented as the mean ± SEM, *n* = 5, and expressed as fold change vs. control group. (**C**) Given the role of Nrf2 and HO-1 in oxidative stress management, levels of 8-hydroxy-2′-deoxyguanosine (8-OHdG) in plasma (left panel) and hepatic tissue (right panel) were assessed 24 h post-ACLF induction by ELISA. Data are presented as mean ± SEM, whereas dots represet individual values, *n* = 8; * *p* < 0.05 vs. control group; & *p* < 0.05 vs. cirrhosis group; # *p* < 0.05 vs. ACLF group.

In this study, we assessed the expression of Nrf2 in the nuclear fraction of hepatic tissue 16 h after acute challenge. No differences were found between the control and cirrhosis groups (Figure S6A); however, samples from the ACLF group exhibited a significant decrease in Nrf2 levels, which was significantly reversed after secretome administration (Figure 5A).

To investigate the downstream cascade of the Nrf2 pathway, the HO-1 protein level was analyzed. No differences were observed between the control and cirrhosis groups (Figure S6B). However, secretome administration significantly increased HO-1 expression 16 h after acute failure induction (Figure 5B).

The generation and accumulation of reactive oxygen species associated with inflammatory processes can lead to oxidative DNA damage, which is related to apoptosis activation [65]. Additionally, it has been reported that patients with liver failure present elevated plasma levels of the DNA oxidation marker 8-hydroxy-2′-deoxyguanosine (8-OHdG), proposed as a marker for liver failure progression [66]. Considering these factors, we evaluated hepatic and plasma levels of 8-OHdG 24 h after acute challenge. As shown in Figure 5C, while no significant differences were found in 8-OHdG levels between the control and cirrhosis groups, animals with ACLF exhibited a significant increase in both hepatic and plasma levels of 8-OHdg, which was prevented by secretome administration.

These results suggest that secretome administration has an effect in inducing the expression of signaling pathways associated with reducing oxidative damage.

### 2.6. MSC-Secretome Administration Mitigates Acute Kidney Injury Associated with ACLF

Systemic inflammation is the primary driver of ACLF occurrence and progression, closely linked to the development of extrahepatic organ failure [8,14].

**Figure 6:**
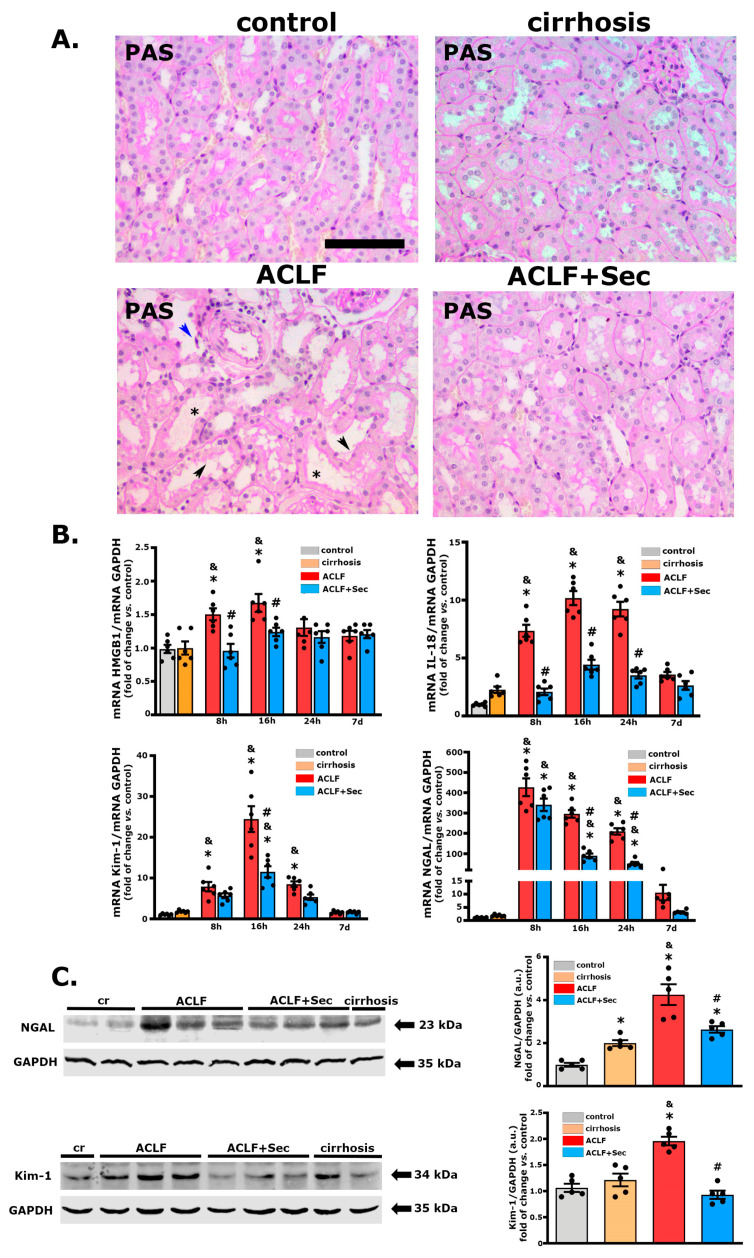
*MSC-secretome administration mitigates acute kidney injury associated with ACLF.* Histological evaluation of renal effects of MSC-secretome administration in ACLF murine model: (**A**) Representative renal tissue micrographs 24 h post-ACLF induction, stained with PAS. ACLF group exhibited histological alterations like brush border loss (black arrows), tubular dilatation (asterisks), and cell detachment (blue arrows). Scale bars represent 100 µm. (**B**) mRNA levels of tubular damage markers HMGB1, IL-18, Kim-1, and NGAL were quantified by RT-qPCR at 8, 16, 24 h, and 7 days post-ACLF induction (LPS/NGalN administration) and normalized against GAPDH expression. Data are presented as mean ± SEM *(n* = 6) and expressed as fold change vs. control group. (**C**) Changes in NGAL and Kim-1 levels were confirmed by Western blotting 16 h post-ACLF induction. Data are presented as mean ± SEM (*n* = 5), whereas dots represet individual values; * *p* < 0.05 vs. control group; & *p* < 0.05 vs. cirrhosis group; # *p* < 0.05 vs. ACLF group.

In this study, the ACLF group treated with vehicle exhibited moderate kidney injury, characterized by pathological alterations, including vacuolization, brush border damage, cell detachment, and tubular dilatation, as evidenced by PAS and H&E staining [67,68]. These morphological alterations, previously associated with AKI development [67], were only subtly present in the secretome-treated animal group (Figure 6A).

Additionally, we assessed the mRNA levels of different markers associated with inflammation and tubular damage, such as IL-18, high-mobility group box 1 (HMGB1), neutrophil gelatinase-associated lipocalin (NGAL), and kidney injury molecule 1 (Kim-1) [69,70,71]. The expression of these markers significantly increased during the acute stage (between 8 and 24 h after ACLF induction) in the ACLF group and returned to normal levels in the surviving animals seven days after the challenge (Figure 6B). In line with the previous results, secretome administration led to a significant decrease in the expression of these molecules. Additionally, changes in NGAL and Kim-1 were confirmed by Western blotting 16 h after ACLF induction (Figure 6C).

Finally, renal function was evaluated by determining the plasma levels of urea and cystatin C (Table 4). Both experimental groups (ACLF and ACLF + Sec) showed a significant increase in plasma urea levels 24 h after the challenge, returning to normal levels seven days later. On the other hand, in the ACLF group, an increase was observed in the plasma levels of cystatin C, which is considered a more sensitive and specific marker related to renal function [72,73], remaining elevated for at least seven days (*p* = 0.064) after acute failure. In contrast, the ACLF + Sec group exhibited a decrease in the plasma levels of cystatin C compared to the vehicle-treated group (Table 4).

Taken together, these results demonstrate that MSC-secretome administration has a renoprotective effect in the studied ACLF model.

### 2.7. MSC-Secretome Administration Reduces the Apoptotic Rate of Renal Cells after ACLF Induction

**Figure 7:**
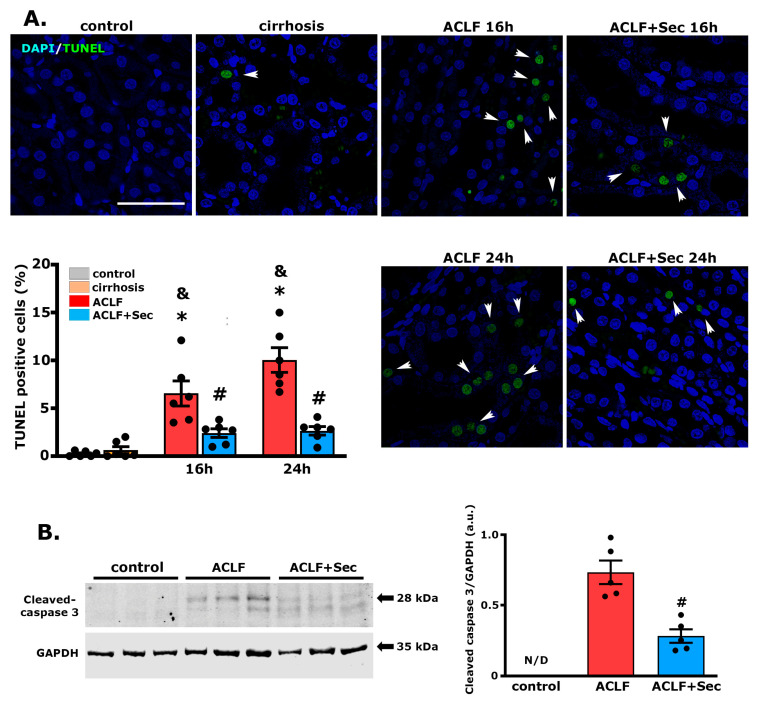
*MSC-Secretome Administration Reduces Apoptotic Rate of Renal Cells Post-ACLF Induction.* MSCs-secretome´s effects on renal apoptosis rate was analyzed at 8, 16 and 24 h post-ACLF induction by TUNEL staining (FITC––green) and confocal microscopy. The nuclei were counterstained with DAPI (blue). (**A**) Representative micrographs of TUNEL-stained apoptosis in tubular sections (white arrowshead). Quantification of positive nuclei per 100 cells was done using digital imaging analysis. Data are presented as mean ± SEM for 30 fields/animal, six animals/group. Scale bars represent 50 µm. (**B**) Complementary apoptosis analysis via Western blot quantifying cleaved caspase-3 levels 16 h post-ACLF induction. Levels were normalized against GAPDH, data are presented as mean ± SEM *n* = 5; whereas dots represents individual values. Expression undetected (N/D) in control group * *p* < 0.05; & *p* < 0.05 vs. cirrhosis group; # *p* < 0.05 vs. ACLF group.

To investigate the effect of secretome on tubular cells further, we assessed the apoptotic rate during the acute phase (between 8 and 24 h after ACLF induction), as previously described for the liver. The ACLF group showed a significant increase in TUNEL (+) cells, while the secretome-treated group exhibited significantly lower values (Figure 7A). These results were confirmed by determining the levels of cleaved caspase 3 in renal tissue 16 h after the acute challenge. In line with the previous result, the levels of this apoptotic marker were virtually undetectable in the control and cirrhosis groups. In contrast, the ACLF group showed a significant increase compared to the secretome-treated group (Figure 7B).

These results demonstrate that the systemic administration of secretome in this ACLF model reduces kidney apoptosis.

### 2.8. MSC-Secretome Administration Reduces Renal Macrophage and Lymphocyte Infiltration

In the context of ACLF, understanding the role of macrophage and lymphocyte infiltration in the progression of renal damage is of utmost importance. The interplay of the hepatic and renal systems is intricate, and immune cell infiltration is a crucial link in the pathophysiological cascade [8].

The infiltration of macrophages has been associated with the initiation and exacerbation of renal damage in ACLF, emphasizing their potential as therapeutic targets to modulate the inflammatory milieu [67]. Simultaneously, the involvement of lymphocytes, particularly T cells, adds another layer of complexity to the interorgan crosstalk since dysregulated immune activation and the infiltration of cytotoxic T cells have been implicated in the progression of renal dysfunction in the context of ACLF [74].

**Figure 8:**
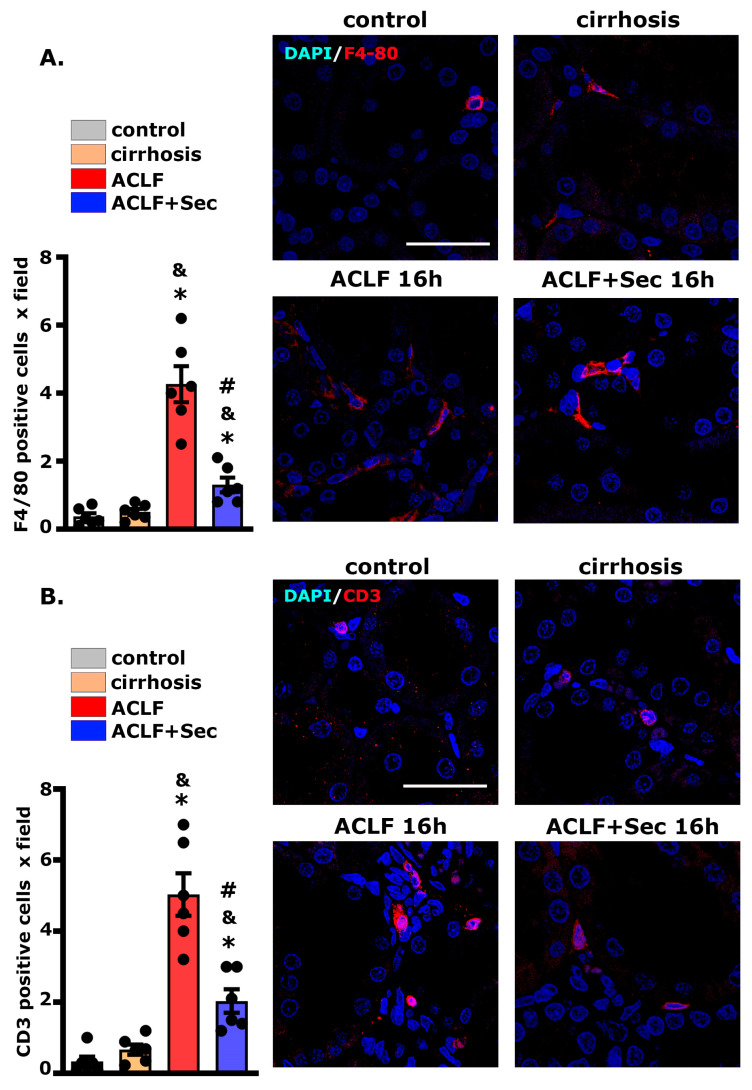
*MSC-Secretome Administration Reduces Renal Macrophage and Lymphocyte Infiltration in the ACLF animal model.* The inflammatory response was assessed by evaluating the infiltration of (**A**) macrophages (F4/80 Alexa 555, red) and (**B**) T lymphocytes (CD3 Alexa 555, red) by confocal microscopy. Nuclei counterstained with DAPI (blue). Scale bars = 50 µm. Quantification of positive cells was carried out using digital imaging analysis. Data are presented as mean ± SEM of 30 fields/animal, six animals/group, whereas dots represet individual values; * *p* < 0.05 vs. control group; & *p* < 0.05 vs. cirrhosis group; # *p* < 0.05 vs. ACLF group.

We employed confocal microscopy to assess macrophage infiltration in the liver 16 h post-acute injury. As shown in Figure 8A, the number of F4/80 (+) cells per field was significantly higher in the ACLF group than in the control and cirrhosis groups. Remarkably, secretome administration induced a marked reduction in the number of macrophages per field. Consistent with the previous result, the number of CD3 (+) cells per field was significantly higher in the ACLF group than in the control and cirrhosis groups. At the same time, the administration of secretome induced a marked reduction in the number of lymphocytes per field (Figure 8B).

Collectively, these findings indicate that the administration of MSC secretome can decrease the renal inflammatory response in the studied ACLF model.

## 3. Discussion

In this study, we employed a previously validated ACLF animal model that mimics the pathophysiological characteristics observed in clinical settings [49,50]. Hepatic cirrhosis was induced by porcine serum administration [75], while the acute precipitating event was simulated by administering LPS/DGalN, mimicking the physiological response to bacterial infections. This model specifically highlights TLR-4-mediated immune cell activation and the subsequent release of proinflammatory cytokines like TNF-α, CINC1, IL-6, and IL-1β, contributing to hepatocyte apoptosis and necrosis [76].

ACLF’s pathological hallmarks include hepatic cell necrosis and inflammatory cell infiltration, leading to hepatic failure. After ACLF induction, we observed an evolution from diffuse to massive necrosis within 16–24 h. TUNEL assessment revealed many hepatocytes undergoing apoptosis, correlating with the mortality observed in ACLF animals. Thus, evaluations were focused on the acute phase (between 8 and 24 h after ACLF induction), with a final follow-up at 7 days, when the hepatic regeneration process typically resolves [58].

MSCs exhibit their most potent therapeutic effects during inflammation, so they should be particularly effective in ACLF. However, clinical studies indicate that while short-term efficacy is favorable, long-term survival did not significantly improve, leading to controversy regarding the benefits of MSC treatment [27,30,77,78,79].

Extensive research highlights the importance of the pathological microenvironment in modulating MSC response, in which aging, immunological, hormonal, and metabolic alterations can impact the therapeutic potential of MSCs [80,81]. In an insightful study, Zhen et al. demonstrated that MSCs exposed to the plasma of ACLF patients lose their immunosuppressive potential and shift toward a pro-inflammatory response [35]. In this study, they proposed that the factors present in the serum of ACLF patients significantly affect the basic metabolism of MSCs, even surpassing the role of some inflammatory factors that can enhance MSCs’ therapeutic effect.

Previous studies emphasize optimizing MSC-based therapies via in vitro preconditioning to boost their therapeutic properties [38]. In that sense, the in vitro culture of MSCs with pro-inflammatory cytokines stimulates their pro-regenerative, anti-inflammatory, and antioxidant capacities [37,41]. Moreover, considering MSCs’ primary action mechanism, namely the secretion of bioactive factors (known as secretomes), the development of cell-free therapeutic strategies has gained significance [40,48].

In the current study, we proposed the use of MSCs derived from human adipose tissue, as they are easily obtainable and abundant, with a higher yield than other sources like bone marrow [82,83]. Their therapeutic effect has been demonstrated in preclinical and clinical models [44,55].

Our approach combined MSC-derived secretome with in vitro preconditioning using TNF-γ and INFγ. This treatment markedly improved 7-day survival rates in ACLF animals, underscoring the enhanced therapeutic potential of the preconditioned secretome.

Considering the frequent use of histopathological analysis in the diagnosis and prognosis of ACLF, our study employed a semiquantitative, validated scoring system to assess liver pathology [55,56]. In ACLF animals, we observed symptoms such as congestion, ballooning, hepatocyte degeneration, extensive necrosis, immune cell infiltration, and significant hepatic architectural distortion. Conversely, animals treated with the secretome displayed a marked reduction in these pathological markers.

Although plasma transaminase levels are used as indicators of hepatic function, in this study, we did not find a correlation between enzyme levels and the survival of animals treated with the secretome. AST and ALT levels significantly increased after ACLF induction, whereas the group treated with the secretome had lower AST levels (16 h post-ACLF induction). The rupture of hepatocyte membranes leads to the release of enzymes into the bloodstream [84]. Since focal necrosis was still observed in animals treated with secretome, a strong correlation between plasma aminotransferase levels and the extent of hepatic damage could not be substantiated. This lack of a relationship between transaminase levels and the degree of hepatic damage has been previously reported in various experimental models and pathological conditions [21,55,85,86].

An increase in direct bilirubin is one of the clinical features of ACLF [3]. Following the administration of MSC secretome, a significant decrease in plasma levels of this metabolite was observed in the treated group. This finding aligns with the reduced congestion hepatic score found in this study, as well as with previous research suggesting that MSC administration can promote bilirubin metabolism [87].

We also investigated the impact of MSC secretome on pro-inflammatory cytokine levels, including TNF-α, CINC1, MCP-1, IL-6, and IL-1β, which are key players in ACLF induction and progression [8]. The increased inflammatory response was only transiently reversed in the case of hepatic TNF-α, IL-6, and CINC1, 16 h post-ACLF induction. Meanwhile, MSC-secretome administration induced a robust anti-inflammatory reaction at both hepatic and systemic levels.

The secretome-induced response could be mediated by a variety of soluble molecules, such as nitric oxide and prostaglandin E2 (PGE2), as well as microvesicles containing different miRNAs (146, 155, 949, 148a, and 1246) that modulate immune cell responses by promoting the secretion of IL-10, IL-5 and IL-4 by dendritic cells and macrophages, which block polymorphonuclear neutrophil influx into the injured tissue and prevent further damage [20,88]. The detailed mechanism through which MSC-secreted factors modulate the immune response remains unclear. Blocking studies have shown that the inhibition of individual factors, including PGED2, IDO, IL-10, and TSG-6, can partially reduce the in vitro and in vivo MSC effects, suggesting that the therapeutic effects of MSCs are mediated by the interaction of various factors that regulate the multiple signaling pathways of the immune system [43,48].

IL-22, a multitarget protein, is unique in the sense that is secreted by immune cells but does not target them [89]. Anti-inflammatory macrophages and dendritic cells primarily produce IL-22 and have been reported to have a hepatoprotective effect via the induction of antiapoptotic activity in diverse hepatic conditions, including ACLF [59,90,91,92]. Its receptor is only expressed in endothelial cells, including hepatocytes [93], but not in immune cells. It is an ideal mediator for tissue repair in ACLF, where the overstimulation of the immune system must be avoided. In agreement with these data, we observed that secretome administration significantly increased the IL-22 receptor in the hepatic tissue, suggesting that this event could be related to the beneficial effects observed after MSC administration.

In hepatic cirrhosis, hepatocytes exhibit increased vulnerability to inflammatory processes and oxidative stress, coupled with a suppressed regenerative capacity, leading to high mortality rates [11,12,59]. In this study, we found that secretome administration after ACLF induction reduced the rate of cellular apoptosis and increased the hepatic regeneration rate. This response could be indirectly mediated through the reduction in the inflammatory response and oxidative stress (see below), in addition to direct mediation by the broad spectrum of molecules present in the secretome, including bFGF, EGF, VEGF, IGF-1, MFGE8, and HGF, with great potential to induce hepatocyte proliferation even in conditions where it is inhibited [21,43,58,94,95].

Nrf2 is a crucial molecule for the modulation of oxidative stress. When Nrf2 enters the nucleus, it binds to the antioxidative response element and promotes the expression of HO-1, an inducible isoform of heme oxygenase, which catalyzes the degradation of heme, producing bilirubin, ferrous iron, and carbon monoxide, which are potent free radical scavengers in the body [96]. Additionally, both factors play an important protective role against inflammatory diseases [97] and induce hepatic regeneration [98,99].

This study suggests that the therapeutic effects of MSC-secretome administration can be mediated through the activation of the Nrf2 pathway; the stimulation of the expression of its target genes, including HO-1; and the translation of this mechanism into a reduction in oxidative damage measured by the hepatic and plasma levels of 8-OHdg. Although multiple studies have shown that MSCs can stimulate the expression of Nrf2 and HO-1, the specific mechanism associated with this effect has not yet been fully elucidated [56,63,100].

Following acute hepatic injury, neutrophils and monocytes are recruited to the liver [101], where they are activated in the context of pro-inflammatory cytokines. Excess infiltration increases tissue damage through increased oxidative damage [102]. In the present study, we observed a decrease in the hepatic infiltration of neutrophils, monocytes, and macrophages, which could be mediated by a reduction in reactive oxygen species resulting from the activation of the Nrf2 pathway [56,103,104].

The direct extension of systemic inflammation to other organs impairs cellular function and may induce necrosis and/or apoptosis. The kidney is highly susceptible to systemic alterations associated with ACLF [105]. The detection of TLR-4 expression in kidney cells indicates a direct link between increasing circulating microbial PAMPs and the subsequent pro-inflammatory cascade in this end-organ injury [105].

The experimental ACLF model used in this study developed extra-hepatic damage, as evidenced by renal alterations. ACLF animals presented moderate tubular histological alterations such as the loss of brush border and tubular dilatation, which have been associated with ACLF-derived kidney injury [67,68]. Furthermore, more sensitive molecular studies revealed an increase in markers of tubular damage and inflammation such as Kim-1, IL-18, HMGB1, and NGAL [69,70,71] in the ACLF group. These changes were associated with a significant increase in the tubular apoptosis rate in the ACLF group. The histological alterations were accompanied by an increase in the plasma levels of urea and cystatin C, both considered reliable markers of kidney function.

Given the pleiotropic effects of secretome bioactive molecules and their systemic administration, treatment could prevent these changes. Additionally, the renal infiltration of macrophages and lymphocytes has been linked to an exacerbation of tissue damage in the context of ACLF [67,74]. Consistent with the hepatic analysis results, the administration of secretome prevented the cellular infiltration of immune cells in the tubular space.

Despite MSCs’ promise, challenges in their clinical application include safety concerns, the need for large cell numbers, and the associated production and handling costs [43,48,74,106,107].

In that sense, interesting previous studies have reported that the administration of living MSCs has a protective effect in a murine model of ACLF. However, the number of cells administered in these reports varied between 2 × 10^6^ and 8 × 10^6^ per animal [50,108]. While these reports are a valuable proof of concept, they present a translational limitation, since in clinical practice, the maximum number of MSCs that can be administered is 2 × 10^6^ per kilogram [109].

In this regard, the boosted secretome used in the present work presents several advantages over the administration of live cells, making it a more promising therapeutic alternative [39]. Moreover, allogeneic MSCs can be obtained from young, healthy donors, who have an advantage in proliferation and anti-inflammatory and pro-regenerative factor production [48]. Finally, MSC secretomes can be lyophilized, facilitating their manufacture their manufacturing, storage, distribution, and administration [110].

In conclusion, in the present work, we found that the systemic administration of MSC-derived secretomes obtained from human adipose tissue that had been boosted in vitro to increase their regenerative capacity had strong hepatic and renal therapeutic effects in a severe ACLF animal model, leading to significantly increased animal survival.

Considering that (i) the mortality of patients with ACLF significantly increases with the involvement of organs other than the liver, most frequently the kidney; (ii) the ineffectiveness of actual approaches seems to be related to the fact that ACLF is a multifactorial triggered disease and involves many interrelated mechanisms; and (iii) the limited time window that patients with ACLF have to receive treatment before suffering severe liver failure, we postulate that the MSC secretome can be a promising alternative for the treatment of these patients.

## 4. Materials and Methods

### 4.1. Isolation, Expansion, and Characterization of MSCs Derived from Human Adipose Tissue

MSCs were isolated from the subcutaneous adipose tissue in the abdominal region and harvested from donors undergoing cosmetic liposuction at Clínica Alemana-Universidad del Desarrollo, Santiago, Chile, as previously described [42]. Written informed consent was obtained from all donors before sample collection. The protocol was approved by the Ethics Committee of the Facultad de Medicina, CAS-UDD. Subsequently, cells were characterized according to their adipogenic and osteogenic differentiation potential by the presence of putative MSC markers (CD29, CD13, CD90, and CD73) and the absence of other cell lineages markers (CD45, CD31, and CD235a), as previously described [42].

### 4.2. MSC Preconditioning and Secretome Generation

The secretome from preconditioned MSCs was harvested following a method previously established by our group [111]. The MSCs derived from human adipose tissue (at passage 3) were incubated in alpha minimal essential medium (α-MEM, Gibco, Carlsbad, CA, USA), supplemented with 10% fetal bovine serum (FBS, HyClone, Marlborough, Australia) and 1.16 mg/mL gentamicin (Sanderson, Laurel, MS, USA) until reaching 70% confluence and preconditioned via incubation with 10 ng/mL TNF-α and 15 mg/mL INFγ (R&D System, Mineapolis, USA) for 40 h. We and others [41,112] have previously described that this preconditioning strategy significantly improves the effects of anti-inflammatory and pro-regenerative factors compared to non-preconditioned MSCs.

After preconditioning, cells were washed with phosphate-buffered saline (PBS, Gibco, Carlsbad, CA, USA) three times and then cultured for an additional 48 h in α-MEM without phenol red and FBS. Subsequently, the culture medium including the secretome was collected and subjected to centrifugation at 400 × *g* for 10 min to separate intact cells. The remaining supernatant was further centrifuged at 5000 × *g* for 10 min to eliminate any cellular debris. This procedure was implemented to minimize the contamination of the secretome with proteins released due to cell rupture. The secretome was then passed through 0.22 µm filters. To concentrate the secretome, they were processed through 3 kDa cutoff filters (Millipore, Burlington, MA, USA), rinsed with 15 mL of PBS, and concentrated again with the same filter type, achieving a 30-fold (*v*/*v*) concentration. The protein concentration was determined using a bicinchoninic acid (BCA) assay kit (#23252, Pierce, Thermo Fisher, Waltham, MA, USA), and the secretome was frozen at −80 °C until use.

### 4.3. Experimental Animals

Sprague Dawley rats weighing 120–150 g were obtained from Universidad de Chile. They were housed in an environmentally controlled room at constant temperature (22 ± 2 °C) and 60% relative humidity, with a 12:12 h light–dark cycle and free access to food and water. All animal protocols were approved by the CICUAL Committee of Universidad del Desarrollo (CICUAL number 6-2020).

### 4.4. Establishment of the ACLF Model

In this study, we employed a previously validated ACLF animal model [49,50,51,52,53,54]. For this, male rats were randomly divided into two groups. The rats in the control group received intraperitoneal (*i.p.*) 0.9% NaCl solution. In contrast, those in the ACLF group were *i.p.*-administered with porcine serum (PS) (P9783 Sigma, Saint Luis, MI, USA) at a dose of 0.5 mL twice weekly for 11 weeks to induce immune-mediated liver fibrosis. After this period, the animals were intravenously injected with LPS (L2880 Sigma, Saint Luis, MI, USA) at a dose of 50 µg/kg. Thirty minutes later, D-galactosamine (D-GalN) (G0500 Sigma, Saint Luis, MI, USA) was *i.p.*-injected at a dose of 600 mg/kg to induce acute liver failure through chronic liver cirrhosis. One hour later, the ACLF rats were randomly divided into two groups. One received 200 µL of concentrated MSC secretome derived from 1 × 10^6^ preconditioned MSCs (ACLF-secretome group), and the other received 200 µL of 0.9% NaCl solution (ACLF group), via the tail vein. Animals were euthanized at 8, 16, 24 h, and 7 days following the administration of MSC secretome or saline solution.

The ACLF condition is considered distinct from decompensated cirrhosis [2]. In this study, a group of animals was euthanized once hepatic cirrhosis was established (via the administration of porcine serum) before inducing acute failure (the administration of LPS/GalN) (cirrhosis group). This group served as a control group to confirm that the observed alterations were associated with the ACLF model rather than chronic cirrhosis.

Body weight was measured during the study, and the weights of the liver, kidney, and spleen were measured immediately after euthanasia. The weights of these organs were normalized relative to body weight and expressed as mg/g of rat body weight. Plasma was stored at −80 °C until use. The hepatic and renal tissues were preserved for subsequent molecular and histological analyses.

### 4.5. Survival Rate and Biochemical Indices

ACLF rats were monitored for survival for 7 days following ACLF induction. The plasma levels of alanine aminotransferase (ALT), aspartate aminotransferase (AST), alkaline phosphatase, direct bilirubin (DBiL), and albumin were measured using a Selectra Pro S autoanalyzer.

For the assessment of renal function, blood urea nitrogen (BUN) was evaluated using a quantitative colorimetric kit (Urea Assay Kit MAK006, Sigma, Saint Luis, MI, USA). The plasma levels of cystatin C were determined by ELISA (ELR-CystatinC RayBiotech, Pottstown, USA).

### 4.6. Hepatic and Renal Histology and Immunofluorescence Analysis

Sections of the right lobe of the liver and right kidney were removed from each group for pathological examination. Following formalin fixation and paraffin embedding, the tissue samples were sectioned into 4 µm slides and stained with hematoxylin–eosin to evaluate leukocyte infiltration. Masson’s trichrome stain was used to assess collagen deposition and fibrosis, and periodic acid–Schiff (PAS) staining was employed to identify glycogen deposition. The severity of liver injury was assessed using the Suzuki classification [113,114], with slight modifications. The scoring for liver injury severity was as follows: none, 0; within the lower third, 1; within the middle third, 2; and within the highest third, 3. Scores were based on the degree of sinusoidal congestion, periportal, and pericentral inflammation, as well as the necrosis of parenchymal cells. All the stained sections were scored by the same pathologist (B.M.) who was blinded for the treatment regimen. Five liver sections per experimental group were evaluated, and five randomly selected high-power fields were analyzed in each liver section. These scores have been previously reported in papers evaluating related hepatic injuries in murine models [55,56,115].

Confocal microscopy was employed for the semiquantitative analysis of macrophage (F4/80) and T lymphocyte (CD3+) infiltration, as previously described [21]. Tissue sections were blocked using 5% FBS in Tris-buffered saline (TBS) and then incubated with primary antibodies (F4/80 ab74383 and α-SMA ab5694 (Abcam, Fremont, CA, USA), along with CD3 (A0452, Dako, Santa Clara, CA, USA)) in SignalStain diluent (Cell Signaling Technology, Massachusetts, USA). Subsequently, these sections were washed and incubated with secondary antibodies, followed by nuclei counterstaining with DAPI. The tissue sections were analyzed using an Olympus Fluoview FV101 confocal microscope.

For the semiquantitative assessment of lymphocyte and macrophage infiltration, CD3+ and F4/80+ cells were evaluated in 30 random sections from each animal. The results were expressed as the number of positive cells per tissue section [116].

To determine the rate of hepatic proliferation, liver sections were stained for proliferating cell nuclear antigen (PCNA) using the anti-PCNA NB600-1331 antibody (Novus Biologicals, Centennial, CO, USA). This analysis was performed through confocal microscopy, as detailed in a previous study [58].

Apoptosis in hepatic and renal tissues was assessed using the terminal deoxynucleotidyl transferase-mediated dUTP nick end labeling (TUNEL) technique with the DeadEnd™ Fluorometric TUNEL System (Promega, Madison, WI, USA). Following nuclei counterstaining DAPI, the labeling indices were examined via confocal microscopy.

The proliferation and apoptosis rates were quantified by counting PCNA (+) and TUNEL (+) nuclei per 100 cells across 30 high-power fields for each animal, with a total of 6 animals in each experimental group. Cell quantification was conducted using ImageJ 1.52a software [58].

### 4.7. RNA Isolation and Gene Expression Analysis

The mRNA levels of the genes of interest were assessed by RT-qPCR as previously described [58]. Total RNA was purified using TRIzol (Invitrogen Waltham, MA, USA), following the manufacturer’s instructions. Two micrograms of total RNA were used to perform reverse transcription with MMLV reverse transcriptase (Invitrogen, Waltham, MA, USA) and oligo dT primers. The expression levels of CINC-1, IL-13, IL-4, IL-5, IL-6, IL-18, MCP-1, TGFβ2, TNF-α, HMGB1, Kim-1, and NGAL were evaluated using specific primers (Table S1) in a LightCycler 1.5 thermocycler (Roche). Relative quantification was performed using the ΔΔCT method [117]. The mRNA level of each target gene was normalized against the mRNA levels of beta-2 microglobulin (B2M) and glyceraldehyde-3-phosphate dehydrogenase (GAPDH) and expressed as a fold of change relative to the control group.

### 4.8. DNA Oxidative Damage Measurement

The formation of 8-hydroxydeoxyguanosine (8-OHdG), a ubiquitous marker of oxidative stress, was evaluated using the OxiSelect Oxidative DNA Damage ELISA kit (Cell Biolabs, Inc. San Diego, CA, USA), according to the manufacturer’s instructions. The level of 8-OHdG in plasma was determined directly, while for its assessment in hepatic tissue, DNA extraction from the tissues was first performed using DNAzol (Thermo, Waltham, MA, USA), following the manufacturer’s instructions. The levels of 8-OHdG were expressed as ng/mL in plasma and ng/microgram of DNA in the hepatic tissue.

### 4.9. Quantification of Systemic and Hepatic Cytokine Levels

Cytokine levels (TNF-α, CINC-1, IL-6, MCP-1, IL-18, IL-13, IL-17, IL-2, IL-10, IL-13, IL-4, and IL-5) were assessed in 25 µL of plasma, as previously described, using the Milliplex MAP Rat Cytokine/Chemokine Magnetic Bead Panel (RECYTMAG-65k, Merk Rahway, NJ, USA), following the manufacturer’s instructions [118]. Protein extraction was performed using a T-PER Tissue Protein Extraction buffer (#78510 Thermo) supplemented with protease inhibitors (Halt Protease Inhibitor Cocktail #78429, Thermo) to evaluate cytokine levels in hepatic tissue. For the assessment, 100 µg of protein per well was utilized.

### 4.10. Quantification of Biological Markers by Western Blotting

Samples were taken from the right lobe of the liver and renal pole of animals from each experimental group. Proteins were extracted using a T-PER Tissue Protein Extraction buffer (#78510, Thermo, Waltham, MA, USA) supplemented with protease inhibitors (Halt Protease Inhibitor Cocktail #78429, Thermo).

For the evaluation of Nrf2 levels, cytoplasmic and nuclear protein extracts were obtained using a Nuclear Extraction kit (#10009277, Cayman, Michigan, USA), as previously described, following the manufacturer’s instructions [63]. The protein concentration was determined using a bicinchoninic acid (BCA) assay kit (#23252, Pierce, Thermo, Waltham, MA, USA).

Western blots were performed using 50 µg of proteins. The membranes were sequentially incubated with appropriate primary and secondary antibodies (Table S2). For the loading control, the membranes were assessed for GAPDH or Histone H4. Reactive bands were detected using the Odyssey Imaging System (LI-COR) and quantified using Image Studio Lite 5.2 software.

### 4.11. Isolation and Flow Cytometric Analysis of Leukocytes

Rats were euthanized with a lethal dose of ketamine (50 mg/kg) and xylazine (5 mg/kg). Blood was obtained via cardiac puncture, and then the animals were perfused with perfusion buffer (dextrose 4%, NaCl 8%, and saccharose 0.8%). After perfusion, the liver sample was collected.

Single-cell suspensions were obtained from the liver samples by performing enzymatic digestion in an RPMI 1640 medium supplemented with 10% FCS, collagenase D 1 (mg/mL) (Roche, Rotkreuz, Switzerland), and DNase I 50 μg/mL (Roche) at 37 °C for 30 min, followed by dissociation with gentleMACS (Miltenyi Biotec, Waltman, MA, USA). Homogenates were passed through a metal mesh. Subsequently, leukocytes were isolated by density gradient centrifugation using 37% Percoll (GE Healthcare Bio-Sciences, Spain).

Cells were surface-stained for 30 min at 4 °C with FITC-conjugated mouse anti-rat Granulocytes, PECy7-conjugated mouse anti-rat CD45, PE-CF594-conjugated mouse anti-rat RP-1, BV786-conjugated mouse anti-rat macrophage subset (all BD Pharmingen), and a fixable dead cell stain (eBioscience™ Fixable Viability Dye eFluor™ 780) to exclude dead cells from analysis.

Samples were measured with a BD FACSAria III (BD Biosciences), and data analysis was performed using the FLowJo V10 software (Treestar, USA) or the FACSDiva Version 6.1.3 (BD Bio-Sciences, USA).

### 4.12. Statistical Analyses

Statistical analyses were conducted using GraphPad Prism 8 software. Data are presented as mean ± SEM. Kruskal–Wallis followed by Dunn’s multiple comparison test as a post hoc test was performed for the comparison of experimental groups. A significance level of *p* < 0.05 was considered statistically significant.

## Figures and Tables

**Table 1:** MSC-secretome administration induces a decrease in liver and spleen index indices after ACLF induction.

			8 h		16 h		24 h		7 Days	
	Control	Chronic	ACLF	ACLF + Sec	ACLF	ACLF + Sec	ACLF	ACLF + Sec	ACLF	ACLF + Sec
liver/body weight ratio (mg/g)	32.16 ± 0.6	35.3 ± 2	40.3 ± 2.1	37.6 ± 1.5	41.6 ± 1.6 a,b	35.8 ± 1.3 a,c	37.9 ± 0.8	38.1 ± 0.32	33.7 ± 0.7	31.7 ± 0.8
kidney/body weight ratio (mg/g)	3.14 ± 0.05	3.41 ± 0.13	3.49 ± 0.14	3.64 ± 0.16	3.50 ± 0.21	3.60 ± 0.19	3.55 ± 0.12 a,b	3.50 ± 0.8	3.38 ± 0.7	3.38 ± 0.17
spleen/body weight ratio (mg/g)	1.69 ± 0.06	2.11 ± 0.15 a	2.89 ± 0.13 a,b	2.75 ± 0.15 a,b	3.18 ± 0.24 a,b	2.5 ± 0.11 a,b,c	3.21 ± 0.41 a,b	2.56 ± 0.3	2.74 ± 0.03	1.91 ± 0.18 c

Liver function in each experimental group was assessed through serological tests. Data are presented as mean ± SEM, *n* = 10; a *p* < 0.05 vs. control group; b *p* < 0.05 vs. cirrhosis group; c *p* < 0.05 vs. ACLF group.

**Table 2:** The biochemical profile after ACLF induction.

			8 h		16 h		24 h		7 Days	
	Control	Cirrhosis	ACLF	ACLF + Sec	ACLF	ACLF + Sec	ACLF	ACLF + Sec	ACLF	ACLF + Sec
AST (U/L)	148 ± 42	243 ± 53	7450 ± 984 a,b	7317 ± 3920 a,b	28,700 ± 3700 a,b	5245 ± 4505 a,b,c	3700 ± 3150 a,b	5233 ± 2648 a,b	112 ± 20	120 ± 35
ALT (U/L)	64 ± 4	86 ± 13	5563 ± 874 a,b	6350 ± 3242 a,b	13,850 ± 3750 a,b	14,071 ± 5862 a,b	5461 ± 5370 a,b	7233 ± 4638 a,b	79 ± 10	73 ± 16
alkaline phosphatase (U/L)	288.8 ± 16	350.2 ± 25	362 ± 39.7	434 ± 31.1	463.8 ± 57.9 a,b	498.3 ± 52.4 a,b	580 ± 98.9 a,b	512 ± 48 a,b	278 ± 28	290.2 ± 41.3
direct bilirubin (mg/dL)	0.06 ± 0.01	0.09 ± 0.01	0.51 ± 0.09 a,b	0.47 ± 0.12 a,b	1.51 ± 0.33 a,b	0.71 ± 0.23 a,b,c	1.81 ± 0.72 a,b	0.42 ± 0.2 a,b,c	0.08 ± 0.02	0.10 ± 0.01
albumin (g/dL)	3.27 ± 0.04	3.20 ± 0.07	3.03 ± 0.05	2.90 ± 0.09	3.40 ± 0.14	3.28 ± 0.11	3.13 ± 0.03	3.07 ± 0.15	3.08 ± 0.06	3.20 ± 0.08

Liver function in each experimental group was assessed through serological tests. Data are presented as mean ± SEM, *n* = 10; a *p* < 0.05 vs. control group; b *p* < 0.05 vs. cirrhosis group; c *p* < 0.05 vs. ACLF group.

**Table 3:** MSC-secretome administration increases hepatic and plasmatic anti-inflammatory cytokine levels post-ACLF induction.

Hepatic Cytokine Levels								
			16 h		24 h		7 Days	
	Control	Cirrhosis	ACLF	Sec	ACLF	Sec	ACLF	Sec
TNFα (pg/mg)	5.1 ± 1.6	5.3 ± 0.9	11.1 ± 0.4 a,b	15.4 ± 1.5 a,b,c	9.9 ± 1.7 a	7.2 ± 1.9	4.8 ± 1.6	4.1 ± 1
CINC-1 (pg/mg)	N/D	170 ± 30	309 ± 110 a	154 ± 33 a,c	140 ± 5 a	129 ± 13 a	160 ± 15 a	130 ± 18 a
IL-6 (pg/mg)	1217 ± 38	790 ± 160	3329 ± 1000 a,b	1271 ± 60 c	853 ± 85	576 ± 51	630 ± 70	680 ± 62
MCP-1 (pg/mg)	44.3 ± 3	36.4 ± 3.3	158.1 ± 32	136.3 ± 25.8	108.9 ± 34.4	83.2 ± 20.5	63.8 ± 15	37.5 ± 8
IL-18 (pg/mg)	471 ± 52	779 ± 200	690 ± 199	946 ± 150	1378 ± 180 a,b	18,822 ± 200 a,b	670 ± 150	638 ± 80
IL-1b (pg/mg)	164 ± 7	183 ± 3	1456 ± 452 a,b	1816 ± 475 a,b	547 ± 151	691 ± 60	200 ± 40	230 ± 18
IL-2 (pg/mg)	45.2 ± 3.5	33.8 ± 6.4	13.4 ± 0.8	39.2 ± 3 c	13.2 ± 2.6	34 ± 5.7 c	25 ± 8	35 ± 7
IL-10 (pg/mg)	133 ± 8	116 ± 16	95 ± 12	111 ± 17	94 ± 16	116 ± 18	78 ± 14	120 ± 18
IL-13 (pg/mg)	5.6 ± 0.1	4.9 ± 0.5	4.3 ± 0.4	7.5 ± 0.5 a,b,c	4.9 ± 0.6	7.3 ± 0.4 a,b,c	3.8 ± 0.4	5.6 ± 0.7
IL-4 (pg/mg)	53 ± 3.5	40 ± 3.7	35 ± 3	52 ± 0.7 b,c	33.5 ± 2.1	55.5 ± 4 b,c	30 ± 1.5	38 ± 8
IL-5 (pg/mg)	24.3 ± 2.9	22.9 ± 1	33.6 ± 2.5	65.1 ± 4 a,b,c	30.4 ± 4.4	58.4 ± 3.8 a,b,c	18 ± 1	25 ± 3
**Plasma Cytokine Levels**								
			**16 h**		**24 h**		**7 Days**	
	**Control**	**Cirrhosis**	**ACLF**	**Sec**	**ACLF**	**Sec**	**ACLF**	**Sec**
TNFα (pg/mL)	1.2 ± 0.2	1.4 ± 0.1	76.3 ± 14.3 a,b	59 ± 22.7 a,b	9.13 ± 5.7 a,b	15 ± 6.2 a,b	6.1 ± 2.7	1.8 ± 0.5
CINC-1 (ng/mL)	N/D	N/D	4 ± 0.3 a,b	3.6 ± 0.2 a,b	2.1 ± 1 a,b	1.8 ± 1 a,b	N/D	N/D
IL-6 (ng/mL)	0.06 ± 0.001	0.07 ± 0.001	11.1 ± 6 a,b	28.6 ± 15.6 a,b	17.2 ± 1 a,b	14 ± 1 a,b	0.1 ± 0.002	0.08 ± 0.025
MCP-1 (ng/mL)	0.032 ± 0.001	0.3 ± 0.09 a	192.5 ± 36.2 a,b	90 ± 23.1 a,b	27.5 ± 10 a,b	41.5 ± 14 a,b	0.2 ± 0.04 a	0.3 ± 0.05 a
IL-17 (pg/mL)	4.6 ± 1	6.7 ± 0.8	59.3 ± 15 a,b	74.6 ± 20 a,b	17.8 ± 12 a,b	29 ± 8 a,b	24 ± 4.7 a,b	18 ± 3.8 a,b
IL-2 (pg/mL)	200 ± 59	50 ± 16 a	226 ± 130	1160 ± 400 a,b,c	160 ± 55	1475 ± 738 a,b,c	83 ± 30	53 ± 15
IL-10 (pg/mL)	236 ± 115	10 ± 0.1 a	591 ± 270 b	1880 ± 620 a,b,c	909 ± 465 a,b	1024 ± 127 a,b	20 ± 0.5	160 ± 32 b
IL-13 (pg/mL)	2.38 ± 0.1	2.38 ± 0.1	5.2 ± 1.8	30.2 ± 12.8 a,b,c	12.3 ± 5.4 a,b	14.3 ± 6 a,b	3 ± 0.2	3.5 ± 0.2
IL-4 (pg/mL)	3.9 ± 0.01	3.9 ± 0.01	5.1 ± 1.1	51.5 ± 2.1 a,b,c	7.8 ± 2.5	4.9 ± 1	3.7 ± 0.1	4.2 ± 0.1
IL-5 (pg/mL)	21 ± 9	19 ± 5	104 ± 3 a,b	194 ± 35 a,b,c	62 ± 30	85 ± 28 a,b	20 ± 6	23 ± 5

Hepatic (upper panel) and plasmatic (lower panel) levels of pro- and anti-inflammatory cytokines were assessed by Milliplex. Remarkable changes between ACLF and ACLF + Sec groups were highlighted. Data are presented as mean ± SEM, *n* = 6; a *p* < 0.05 vs. control group; b *p* < 0.05 vs. cirrhosis group; c *p* < 0.05 vs. ACLF group. N/D: not detected.

**Table 4:** MSC-secretome administration prevents renal failure after ACLF induction.

			24 h		7 days	
	Control	Cirrhosis	ACLF	Sec	ACLF	Sec
plasma urea (ng/µg)	0.551 ± 0.039	0.651 ± 0.069	0.993 ± 0.088 a,b	1.1 ± 0.108 a,b	0.731 ± 0.07	0.515 ± 0.009
plasma cystatin (ng/mL)	17.3 ± 1.6	16.2 ± 2.8	70.0 ± 18.4 a,b	35.1 ± 7.1 c	42.2 ± 15.5	14.7 ± 2.1

Plasmatic markers of renal function urea and cystatin C were evaluated 24 h and 7 days post-ACLF induction. Data are presented as mean ± SEM, *n* = 8; a *p* < 0.05 vs. control group; b *p* < 0.05 vs. cirrhosis group; c *p* < 0.05 vs. ACLF group.

## Data Availability

Data are contained within the article and Supplementary Materials.

## Supplementary Materials

The following supporting information can be downloaded at: https://www.mdpi.com/article/10.3390/ijms25042073/s1.
