# Supplementary material for: The Immunoregulatory and Regenerative Potential of Activated Human Stem Cell Secretome Mitigates Acute-on-Chronic Liver Failure in a Rat Model
Source: Int J Mol Sci. 2024 Feb 8;25(4):2073. doi: 10.3390/ijms25042073 (PMC10889754; doi:10.3390/ijms25042073)
Supplement: Supplementary file 1 [file ijms-25-02073-s001.zip › Supp Table 2.pdf]

Supp Table S2. Antibodies utilized for western blott and immnunofluorescence

IL-6	Santa Cruz Biotec	SC 57315	Mouse Monoclonal	21 kDa
NGAL	Santa Cruz Biotec	SC 515876	Mouse Monoclonal	23 kDa
HMG-1	Santa Cruz Biotec	SC56698	Mouse Monoclonal	30 kDa
PCNA	Santa Cruz Biotec	SC56	Mouse Monoclonal	35 kDa
HISTONA H4	Santa Cruz Biotec	SC377520	Mouse Monoclonal	11 kDa
GAPDH	Santa Cruz Biotec	SC32233	Mouse Monoclonal	36 kDa
IL-18	Novus Biologicals	NBP3-03699	Rabbit Policlonal	22 kDa
KIM-1	Novus Biologicals	NBP1-76701	Rabbit Policlonal	34 kDa
CAS-3 CLEAVED	Cell Signaling Tech	#9661	Rabbit Policlonal	17-19 kDa
GAPDH	Cell Signaling Tech	#2118S	Rabbit Monoclonal	37 kDa
HO-1	Cell Signaling Tech	#43966	Rabbit Monoclonal	28 kDa
NFR2	Cell Signaling Tech	#33649	Rabbit Monoclonal	130 kDa
ALPHA-SMA	ABCAM	ab5694	Rabbit Policlonal	42 kDa
