# Supplementary material for: The Immunoregulatory and Regenerative Potential of Activated Human Stem Cell Secretome Mitigates Acute-on-Chronic Liver Failure in a Rat Model
Source: Int J Mol Sci. 2024 Feb 8;25(4):2073. doi: 10.3390/ijms25042073 (PMC10889754; doi:10.3390/ijms25042073)
Supplement: Supplementary file 1 [file ijms-25-02073-s001.zip › Supp. Table 1.pdf]

Supp Table S1. RT-qPCR specific primers and characteristics of amplicons.

Gene	Nº GenBank access	Sense (5'→3')	Antisense (5'→3')	Amplicon	
				size (bp)	Tm (ºC)
rat CINC-1	NM_030845.2	CCATTAAGTGTCAACCACTGTGCTA	CACATTTCCTCACCCTAACACAAA	100	84
rat IL-13	NM_053828.1	GCAACATCACACAAGACCAGA	GTCAGGGAATCCAGGGCTAC	233	88
rat IL-4	NM_201270.1	ATGCACCGAGATGTTTGTACC	TTTCAGTGTTCTGAGCGTGGA	238	88
rat IL-5	NM_021834.1	TGCTTCTGTGCTTGAACGTTCTAAC	TTCTCTTTTTGTCCGTCAATGTATTTC	298	86
rat IL-6	NM_012589.2	CCAGTTGCCTTCTTGGGACT	TCTGACAGTGCATCATCGCT	224	86
rat IL-18	NM_019165.2	ACCGCAGTAATACGGAGCAT	CAGTCTGGTCTGGGATTCGT	117	84
rat MCP-1	NM_031530.1	GTGCTGACCCCAATAAGGAA	TGAGGTGGTTGTGGAAAAGA	185	84
rat TGFβ1	NM_021578.2	CACCCGCGTGCTAATGGT	GGCACTGCTTCCCGAATG	111	83
rat TNF-α	NM_012675.3	AAATGGGCTCCCTCTCATCAGTTC	TCTGCTTGGTGGTTTGCTACGAC	111	86
rat HMGB1	NM_012963.3	ATTGCTGCCTACAGAGCTAAA	GTCGTCTTCCTCTTCCTTCTTT	96	83
rat Kim-1	NM_173149.2	TGGCACTGTGACATCCTCAGA	GCAACGGACATGCCAACATA	111	84
Rat NGAL	NM_130741.1	GGGCTGTCCGATGAACTG	CATTGGTCGGTGGGAACAGA	98	84
rat IL-22	NM_001191988.1	CTGCCTGCTTCTCGTTGCT	TAAAGGTGCGGTTGACGATG	116	86
rat IL-22 receptor	NM_001003404.2	GGCAAAGGGATCTCTCTGTTCT	TAGGAGAGGCAGTGTAGTTCGC	143	85
rat GADPH	NM_017008.4	GACATGCCGCCTGGAGAAAC	AGCCCAGGATGCCCTTTAGT	92	87
rat β2 microglobulin	NM_012512.2	GAAGGAGCCCAAAACCGTCA	CCGGATCTGGAGTTAAACTGGT	92	84
