# Supplementary material for: The Immunoregulatory and Regenerative Potential of Activated Human Stem Cell Secretome Mitigates Acute-on-Chronic Liver Failure in a Rat Model
Source: Int J Mol Sci. 2024 Feb 8;25(4):2073. doi: 10.3390/ijms25042073 (PMC10889754; doi:10.3390/ijms25042073)
Supplement: Supplementary file 1 [file ijms-25-02073-s001.zip › Supplementary Figures V2.pdf]

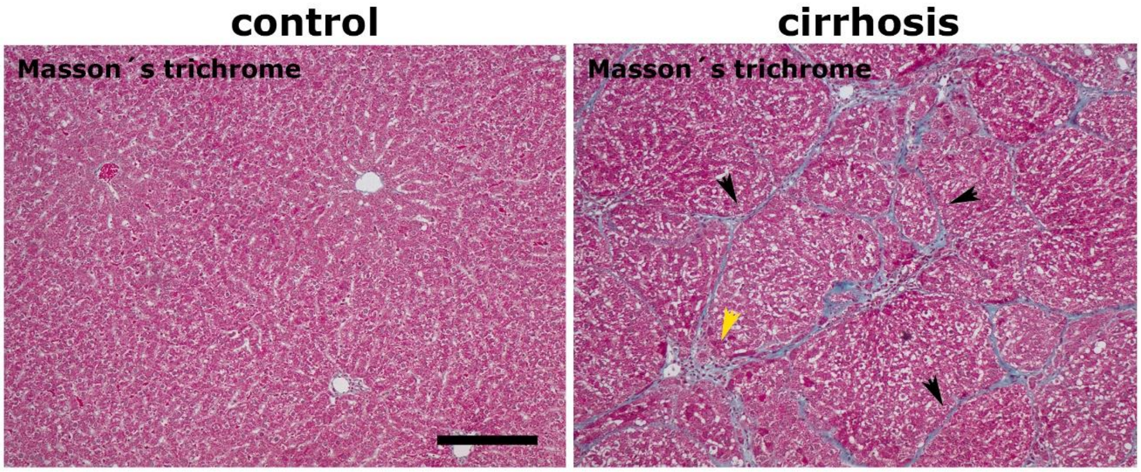

Supplementary Figure S1: *Induction of the chronic component (hepatic cirrhosis) in the murine ACLF model.*

To induce the ACLF model, rats received biweekly porcine serum (SP) administration over 11 weeks, leading to immune-mediated fibrosis, while control animals were injected with the vehicle. Livers from control animals (left panel) exhibited clear structure, uniform hepatocyte sizes, and no microarchitecture distortion. Livers from SP-treated animals (right panel) showed mild inflammatory cell infiltration (yellow arrows), pseudolobule formation, and fibrotic septum development (black arrows). Scale bar represent 250 µm.

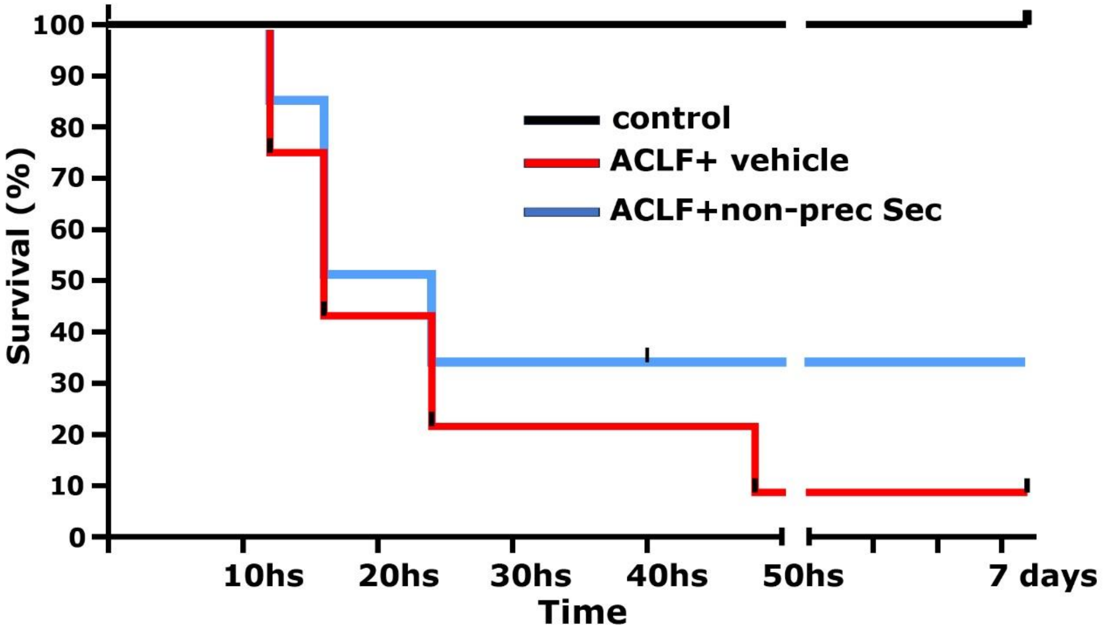

Supplementary Figure S2: *Administration of secretome derived from non-preconditioned MSCs does not increase survival rate in ACLF animals.*

To establish the ACLF model, rats were exposed to porcine serum for 11 weeks (cirrhosis development), followed by an acute challenge with LPS/NGalN (ACLF group). Survival rates were assessed up to 7 days post-challenge. One group of animals was treated with secretome derived from $1x10^6$ non-preconditioned MSCs (ACLF+non-prec Sec), while the other group was treated with the vehicle. Kaplan-Meier survival analysis for control group n=10, ACLF, and ACLF+non prec Sec groups n=35.

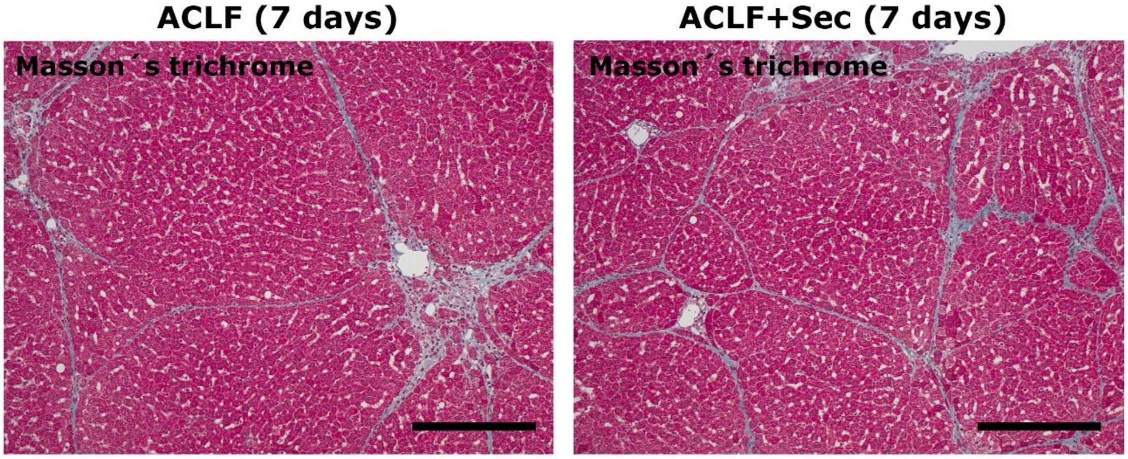

Supplementary Figure S3: *Resolution of hepatic inflammatory and necrotic processes 7 days after ACLF induction.*

Representative micrographs of the hepatic tissue 7 days post-ACLF induction (LPS/DGalN administration). Both experimental groups, ACLF (left panel) and ACLF+Sec (right panel) showed recovery from hepatic microarchitectural alterations, significant reduction in inflammatory activity, and disappearance of apoptosis. Scale bar represent 250 µm.

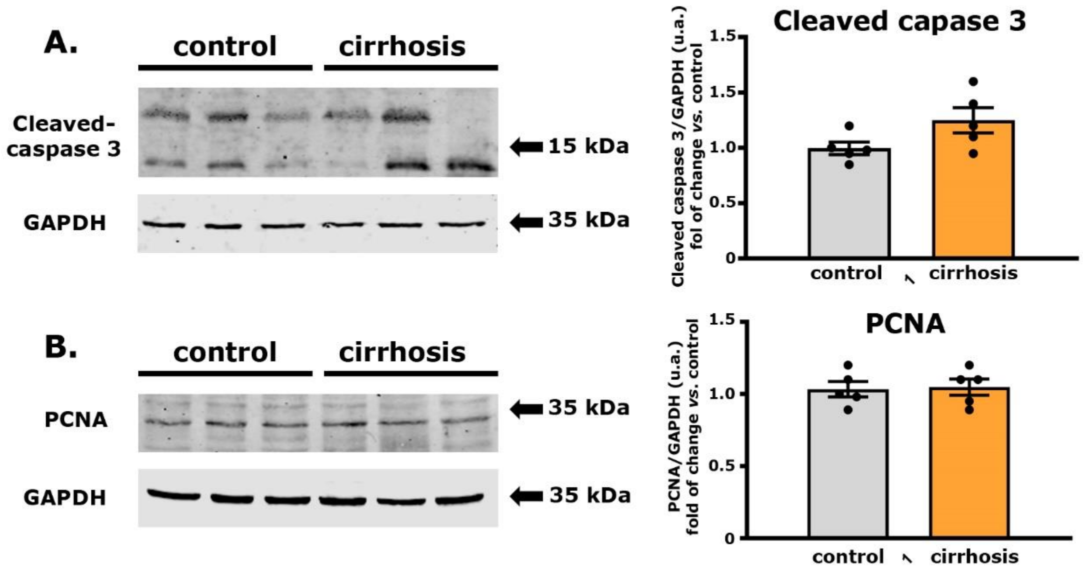

Supplementary Figure S4: *Chronic cirrhosis condition do not induce changes in hepatic apoptosis and proliferation rates.*

(A) Hepatic apoptosis assessed by cleaved caspase-3 levels and **(B)** hepatic proliferation evaluated by PCNA levels via Western blot in the chronic cirrhosis group, induced by porcine serum administration compared to the control group. Protein levels were normalized against GAPDH. Data are presented as mean ± SEM, values were expressed as fold change *vs.* control group. n=5.

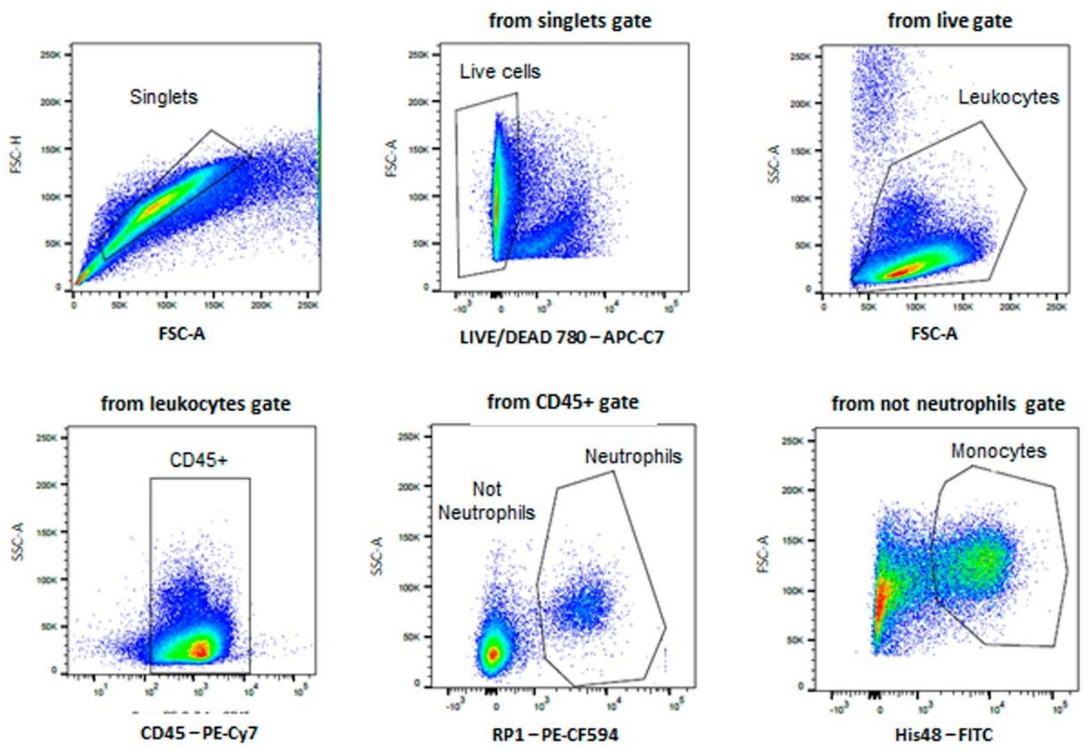

Supplementary Figure S5*: Flow cytometry gating strategy for murine inflammatory cell analysis in the liver.*

Monocytes were distinguished from neutrophils and macrophages based on cell size, complexity, and surface markers. Cells were first selected by singlets, then dead cells were excluded. Cells were separated by size and complexity and then by CD45+ cells. Neutrophils were identified by positive RP-1 marker labeling. All cells not presenting a positive label were analyzed to select monocytes by His48 labeling.

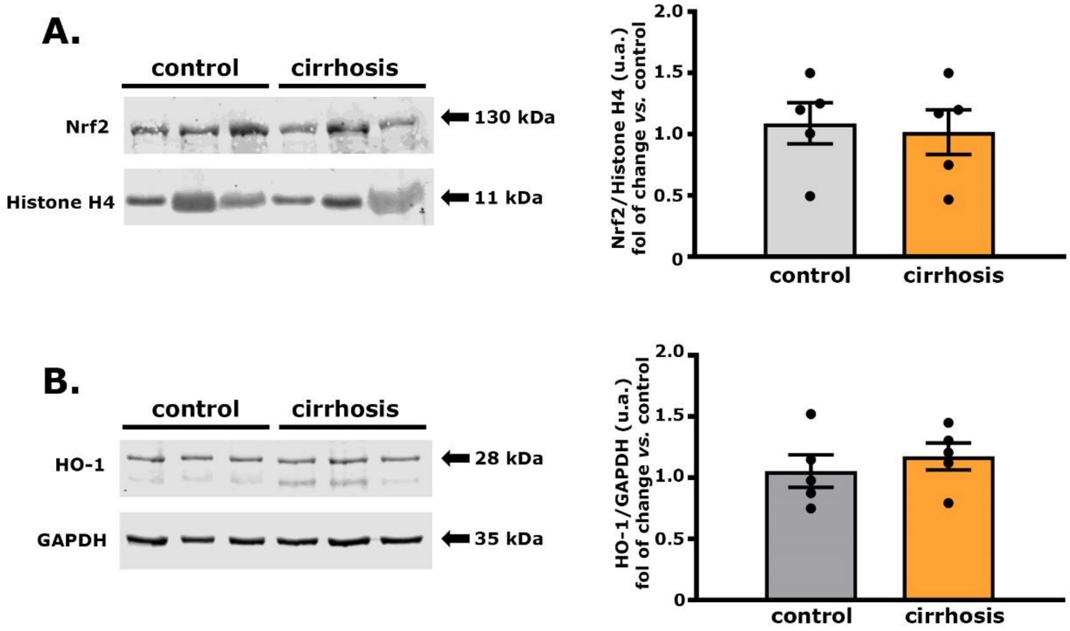

Supplementary Figure S6: *Chronic cirrhosis condition shows no changes in hepatic Nrf2 and HO-1 expression levels.*

Western blot analysis of **(A)** hepatic Nrf2 expression levels in the nuclear fraction of animals treated with porcine serum for 11 weeks to induce chronic hepatic cirrhosis. Values normalized against Histone 4. **(B)** HO-1 levels determined in total liver tissue homogenate. Values normalized against GAPDH. Data are presented as mean ± SEM, values were expressed as fold change *vs.* control group. n=5.
